# Analysis of the active ingredients and health applications of cistanche

**DOI:** 10.3389/fnut.2023.1101182

**Published:** 2023-03-03

**Authors:** Shiqi Zhou, Duo Feng, Yaxi Zhou, Hao Duan, Yongjun Jiang, Wenjie Yan

**Affiliations:** ^1^College of Biochemical Engineering, Beijing Union University, Beijing, China; ^2^Beijing Key Laboratory of Bioactive Substances and Functional Food, College of Biochemical Engineering, Beijing Union University, Beijing, China; ^3^Inner Mongolia Sankou Biotechnology Co., Ltd., Ordos City, Inner Mongolia, China

**Keywords:** Cistanche, homology of medicine and food, active ingredient, PHG, health benefits

## Abstract

Cistanche is a tonic Chinese medicine commonly used in traditional Chinese medicine, with 2016, CFSA through the alxa desert cistanche safety evaluation, cistanche began to officially enter the food field. At present, the research on cistanche mainly focuses on the extraction, isolation and purification and pharmacological effects, and its pharmacological effects such as neuroprotective effects, immunomodulation, antioxidant anticancer and hepatoprotective liver protection have attracted the attention of researchers. This review mainly reviews the research status, chemical composition and health benefits, analyzes its application prospects in food, and aims to provide certain theoretical support for the safe application of cistanche in functional food.

## 1. Introduction

Cistanche (*Cistanche deserticola Ma*) is a perennial parasitic herb of cistanches in the family *Orobanchaceae*, also known as golden shoots, goblins, and brassica, which is mainly produced in China in inner Mongolia, Xinjiang, Ningxia, Gansu, and Qinghai (1). There are about 22 species of cistanche in the world, mostly distributed in dry areas such as warm deserts and deserts in the northern hemisphere. According to the flora of China, there are currently 6 species of cistanche recorded in China. They are Cistanche deserticola (*Citanche deserticola Y.C.M*), Cistanche lanzhouensis (*Cistanche lanzhouensis Z. Y. Zhang*), Cistanche mongolica (*Cistanche mongolica Beck*), Cistanche salsa [*Cistanche salsa (C. A. Mey.) G.Beck*], Cistanche sinensis (*Cistanche sinensis G.Beck*), and Citanche tubulosa [*Citanche tubulosa (Schenk) Wight*] (2). Chinese edible cistanche has a long history, first recorded in the “Shennong Materia Medica” and was listed as a superior product, which has the effect of tonifying kidney yang, improving sperm and blood, and moisturizing the intestines and laxatives.

In 2016, the expert review committee of the China National Center for Food Safety Risk Assessment (CFSA) reviewed in accordance with legal procedures and found that Alxa Desert Cistanche meets food safety requirements based on existing hygienic and toxicological tests and related safety data. In 2018, the desert cistanche was included in the Catalog of Substances That are both food and chinese medicinal materials according to tradition by the National Health Commission, which shows that cistanche can be used as a daily food for the health care of the general population (3). In 2020, the National Health Commission and the State Administration of Market Supervision officially issued a notice on the pilot work on the material management of 9 substances such as party ginseng, which are both food and Chinese medicinal materials in accordance with tradition. And cistanche (*Cistanche deserticola*) is listed among them. Since then cistanche has been officially approved to enter the list of new food raw materials as a medicinal and food homologous substance. At present, China has carried out pilot work on cistanche food and drug substances in Inner Mongolia, Inner Mongolia Ordos, Ningxia Hui Autonomous Region, Gansu and Qinghai, while the published local food safety standards are DBS62/003-2021 in Gansu and DBS63/00016-2021 in Qinghai Province, of which the standard in Qinghai Province stipulates that the daily recommended consumption is 6 to 10 g/d. As of June 29, 2022, there are 60 registered health foods with cistanche, cistanche and cistanche extract as the main raw materials available on the national “special food information inquiry platform.” Their main health care functions are to relieve physical fatigue, regulate immunity and antioxidants, which greatly enriches the sales market of cistanche products.

To put it briefly, cistanche has a variety of nutritional and functional properties, and its application in functional food processing is increasing. At present, the research on the bioactive substances in cistanche is still deepening. This article will review the nutritional and bioactive components of cistanche and its impact on health. So as to better expound the potential impact of cistanche on human health and provide theoretical reference for its safe application in the food field.

## 2. Nutritional value and bioactive compounds of cistanche

With the growing awareness of health care among consumers, this has increased the application of cistanche and its extracts in food. As a food, one of the most important points of consumers is the nutritional value of food. Because health food needs to declare the health function of the product when declaring, it is necessary to understand the bioactive ingredients of cistanche.

### 2.1. Nutritional value of cistanche

Cistanche, as a common Chinese medicinal material for the prescription of Traditional Chinese medicine tonics, weighs, is hard, not easy to break, and is mostly flat cylindrical in shape, slightly curved, and the surface is tan or gray-brown and arranged with wavy rings. In order to further promote the application of cistanche in food, Kurban et al. (4) used burning method, Philin’s method, alkali titration method, Sox extraction method, ultraviolet spectrophotometry to determine the content of general nutrients, vitamin A, vitamin C and cholesterol in cistanche, and also used atomic absorption spectrophotometry to determine the content of trace elements in cistanche. As can be seen from Table 1, cistanche has the highest total acid content and the lowest cholesterol content. Among the elements tested, the macro element Na had the highest content, followed by potassium, the content of manganese, iron, copper and zinc in the essential trace elements was higher, and the content of nickel was the lowest.

**Table 1:** General nutrient and trace element content in cistanche.

Nutrient	Content (per 100 g of original fruit)	Element	Content (μg/g original fruit)
Moisture (g)	7.16	K	7.60 × 10^4^
Fat (mg)	12	Na	1.14 × 10^5^
Cholesterol (μg)	102	Ca	3.63 × 10^4^
Ash (g)	0.24	Fe	1.05 × 10^4^
Dietary fiber (g)	0.19	Mg	9.09 × 10^3^
Reducing sugar (mg)	7.34	Mn	2.02 × 10^2^
Total acid (g)	1.56	Zn	1.07 × 10^2^
V_A_ (mg)	2.42	Cu	9.42 × 10^2^
V_C_ (mg)	3.12	Sr	1.55 × 102
		Ni	11.53

At present, the literature shows that vitamin A, as an essential fat-soluble micronutrient that the human body cannot synthesize, must be obtained from the diet, and V_A_ supplementation can delay cellular aging, promote wound healing, anti-inflammatory, and can also be used for xerophthalmia and blindness prevention (5–7). Vitamin C is a water-soluble vitamin, which can be obtained from fresh fruits and vegetables, is mostly used for preservatives in food to prolong the shelf life of food, can play an antioxidant and strengthen the immune system in the human body, and is clinically used to treat scurvy (6, 8). Dietary fiber is a polysaccharide, and proper intake can help improve intestinal function, promote intestinal health, regulate the body’s immunity, and effectively control blood sugar and blood lipid levels (9). Trace elements are found in smaller amounts in the human body, but play an important role in maintaining the body’s normal physiological functions, such as participating in antioxidant defense, immune response, and wound healing (10). Therefore, cistanche has a high health value in food processing.

### 2.2. Bioactive ingredient of cistanche

So far, the literature has shown that the health value of cistanche is closely related to the bioactive ingredients it contains, and more than 120 compounds have been isolated from cistanche, including phenethyl glycosides, cycloenne ether terpenes and their glycosides, lignans and their glycosides, oligosaccharide esters, polyols and polysaccharides (11, 12). Among them, studies have shown that phenylethanoid glycoside (PhG) extracted from cistanche exceed 80%. As the main active ingredient of cistanche, PhGs have a significant effect on the quality of vascular dementia, and play a positive role in the prevention and treatment of Alzheimer’s disease (AD) (13). Cistanche polysaccharides have a positive effect on immune regulation and can play an anti-peroxidant role in the body (14).

#### 2.2.1. Phenylethanoid glycosides

PhGs are currently the most studied class of compounds among the active ingredients of cistanche, as one of the main components of cistanche, it plays an active role in antioxidants, protecting liver, myocardium, nerve cells and enhancing memory (15, 16). At present, Zhang et al. (17) established a novel HPLC-LTQ-Orbitrap-based strategy, 69 PhGs (LTQ) isolated from cistanche and cistanche. Li et al. (18) used LC/QTOF-MS/MS to find 21 phenethyl alcohol glycosides in cistanche. Ai et al. (19) found 10 PhGs by using UHPLC–MS/MS. Li et al. (20) used quantitative analysis of multi-components by single marker (QAMS) to determine the content of 5 phenethyl alcohol glycosides in 10 batches of cistanche, providing an economical and reliable method for quality control. Yan-xia et al. (21) and Lu et al. (22) used high performance liquid chromatography(HPLC) method to determine the phenethyl alcohol glycoside compounds in cistanche. It was clear that the phenethyl alcohol compounds of different types of medicinal materials were different. And Jian-song et al. (23) used ultra performance liquid chromatography(UPLC) method to determine 6 phenethyl alcohol glycoside components in cistanche, which provided an obvious basis for the quality evaluation of genuine and counterfeit products. Besides Xie et al. (24) used a solvent system ofethyl acetate-n-butanol-glacial acetic acid-water (1, 1.2, 0.2, 2, v = v = v = v) to isolate and purify echinacoside and acteoside. And Yang et al. (25) established PhGs chemical fingerprints of cistanche from different regions of China. At present, through literature search, it has been found that more than 70 PhGs can be isolated, including 4 monoglycosides, 41 disaccharides and 25 triglycosides (Table 2). It can be concluded that the study of the purification and extraction of the active ingredient of PhGs in cistanche. They are helpful to the study of the medicinal value of cistanche and also have guiding value for the study of the mechanism of action of the body.

**Table 2:** Phenylethanoid glycosides from cistanche species.

Chemical structure	PhGs compound	Ref	Chemical structure	PhGs compound	Ref
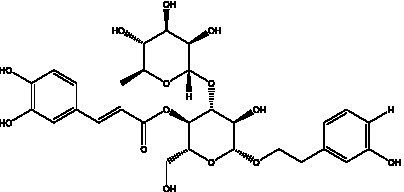	acteoside	(26)	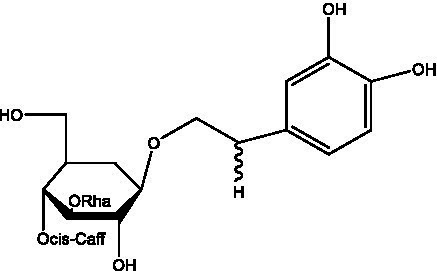	Cis-acteoside	(27)
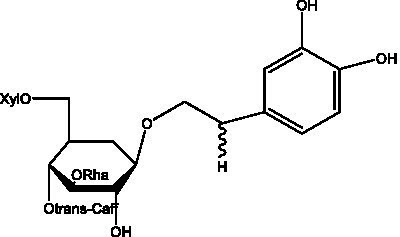	arenarioside	(27)	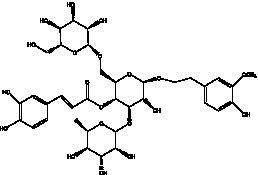	Cistanoside A	(26, 28)
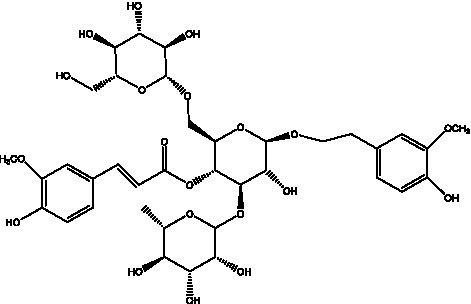	Cistanoside B	(28, 29)	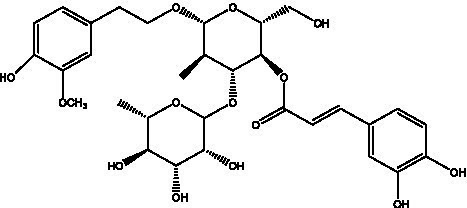	Cistanoside C	(26, 30)
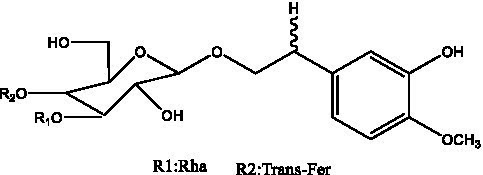	Cistanoside D	(30)	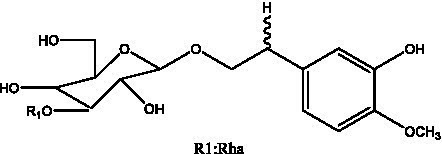	Cistanoside E	(28)
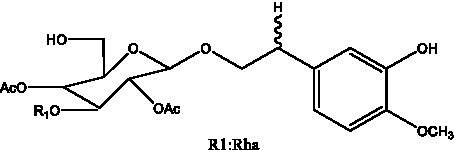	Cistanoside F	(31, 32)	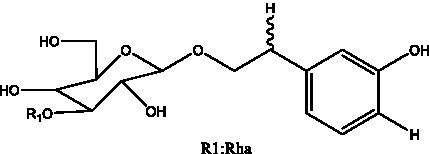	Cistanoside G	(32)
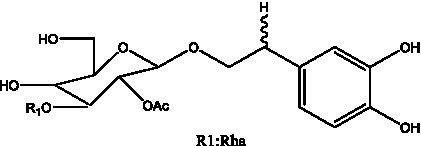	Cistanoside H	(32)	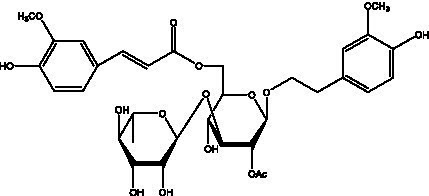	Cistanoside J	(33)
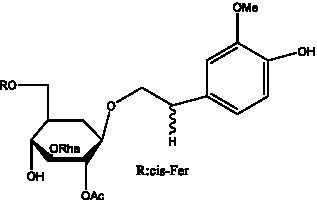	Cis-cistanoside J	(34)	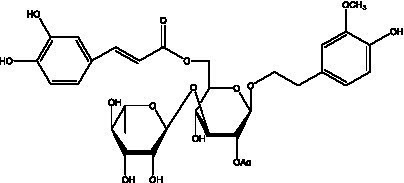	Cistanoside K	(33)
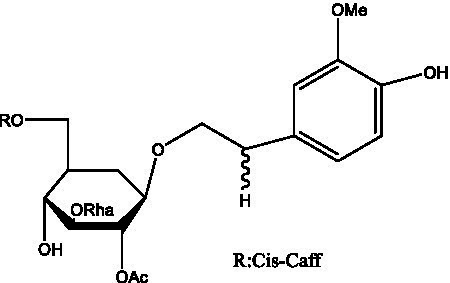	Cis-cistanoside K	(12)	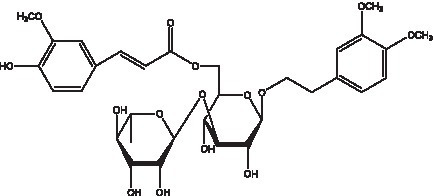	Cistanoside L	(33)
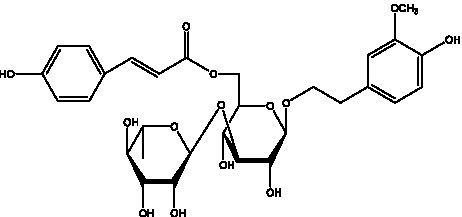	Cistanoside M	(33)	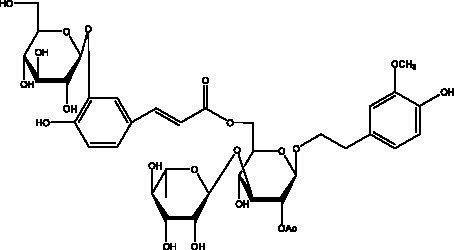	Cistanoside N	(33)
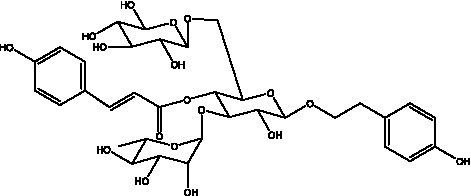	Cistantubuloside A	(31)	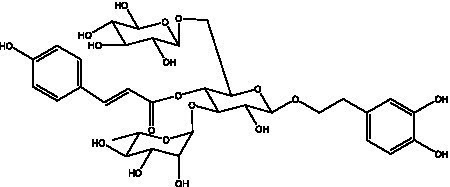	Cistantubuloside B1	(31)
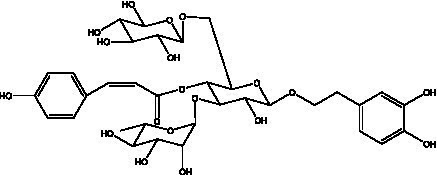	Cistantubuloside B2	(31)	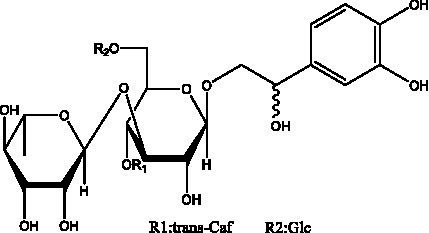	Cistantubuloside C1/C2	(31)
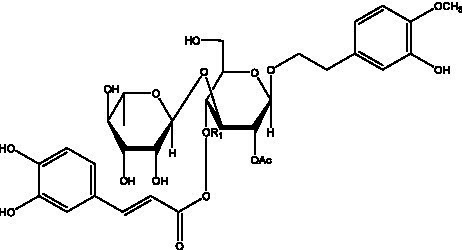	Cistansinenside A	(35)	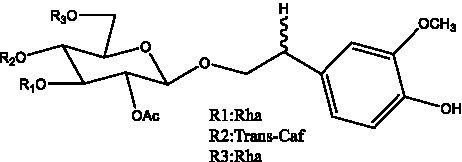	Cistansinenside B	(35)
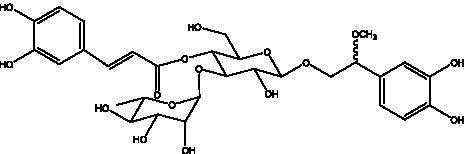	Campneoside I	(36)	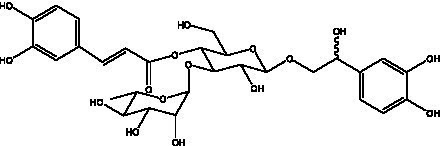	Campneoside II	(36)
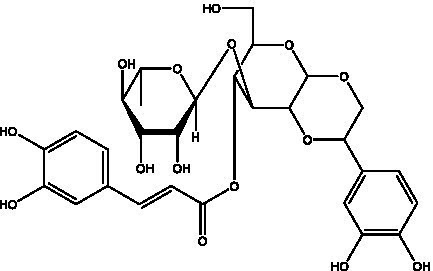	Crenatoside	(27)	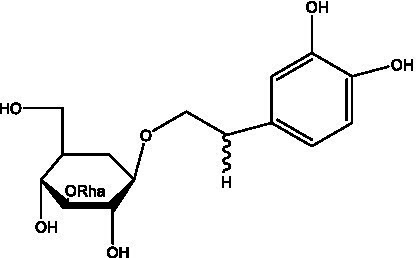	Decaffeoylacteoside	(37)
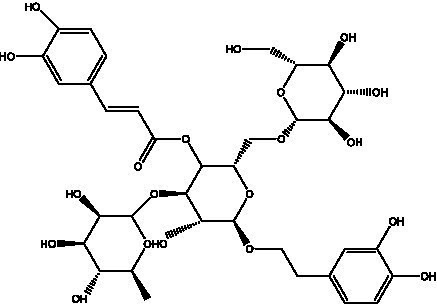	Echinacoside (ECH)	(26)	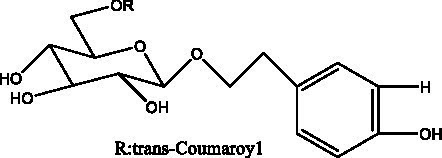	Eutigoside A	(38)
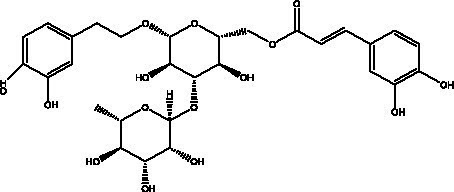	Isoacteoside	(26)	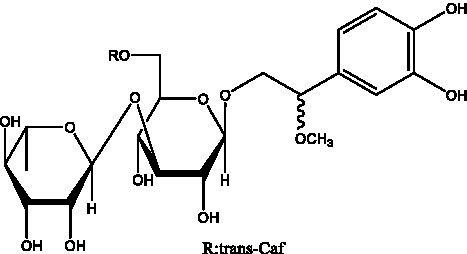	IsocampneosideI	(36)
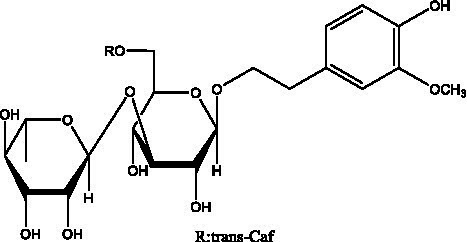	Isocistanoside C	(39)	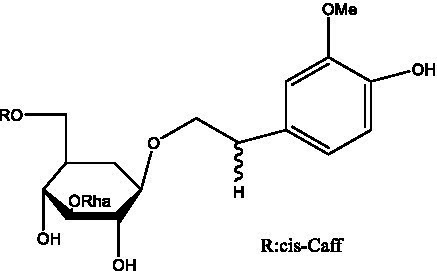	cis-isocistanoside C	(12)
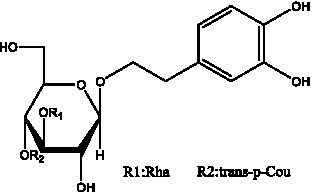	Isosyringalide A 3′-α-L-rhamnopyronoside	(39)	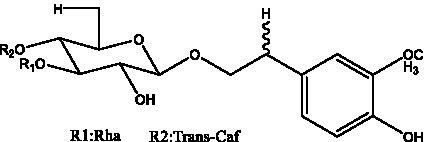	Jionoside D	(40)
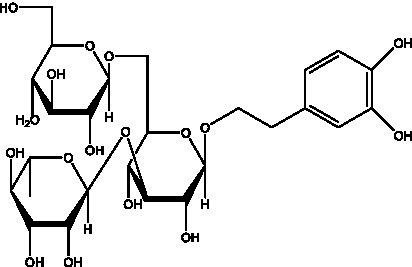	Kankanoside F	(32, 33)	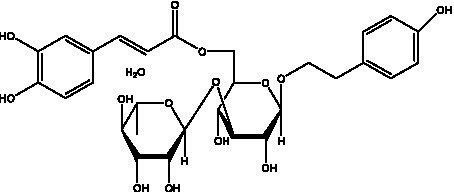	Kankanoside G	(32)
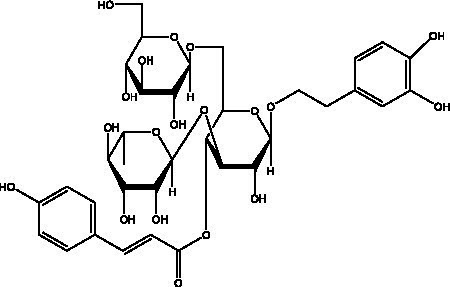	Kankanosides H_1_	(27)	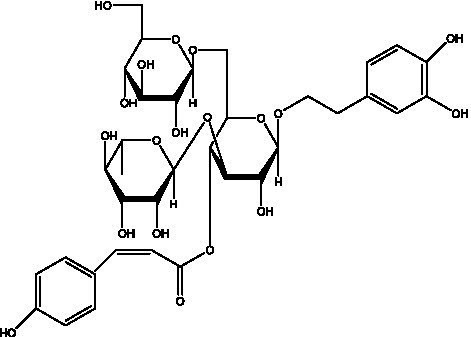	Kankanosides H_2_	(27)
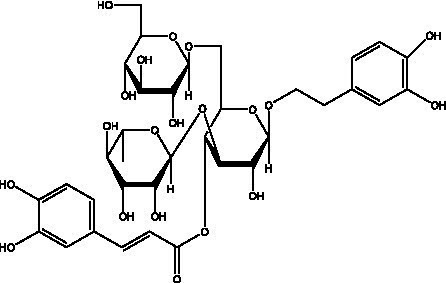	Kankanosides I	(27)	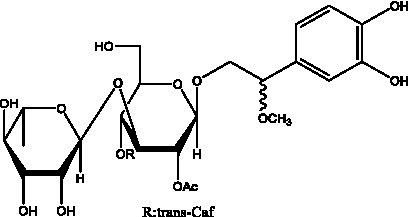	Kankanosides J1/ J2	(36)
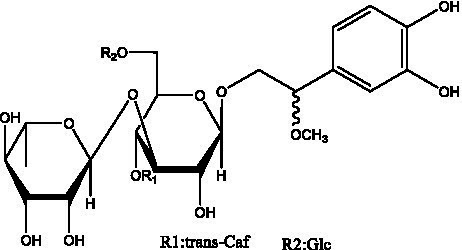	Kankanosides K1/ K2	(36)	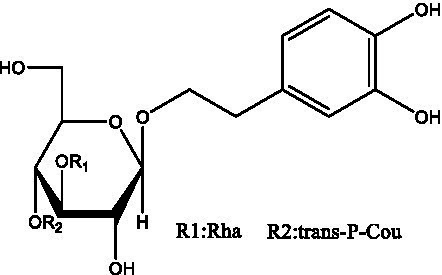	Osmanthuside B	(41)
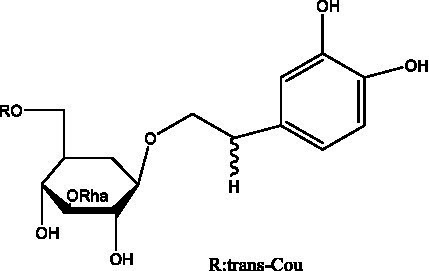	Osmanthuside B6(E)	(12)	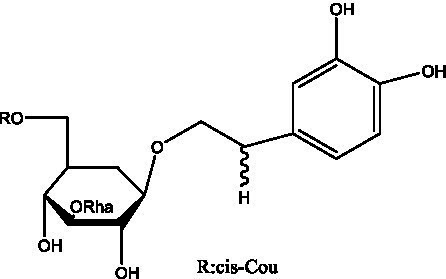	Osmanthuside B6(Z)	(26)
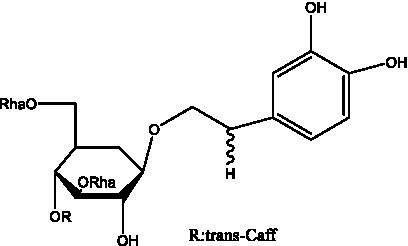	Plantainoside C	(34)	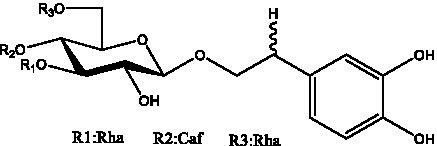	Poliumoside	(40, 41)
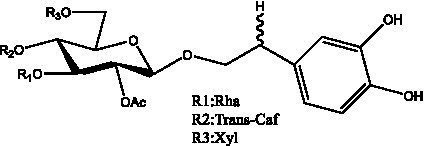	Pheliposide	(11)	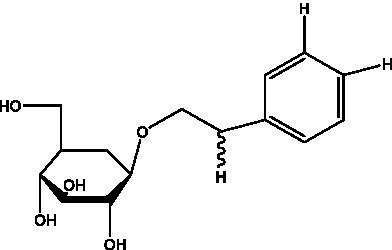	Phenylethyl-glucopy ranoside	(34)
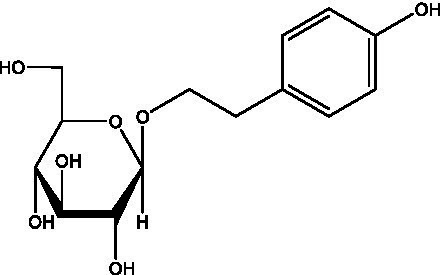	Salidroside	(32)	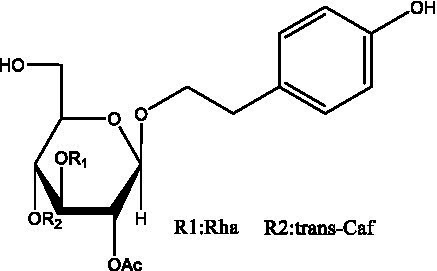	Salsasides D	(42)
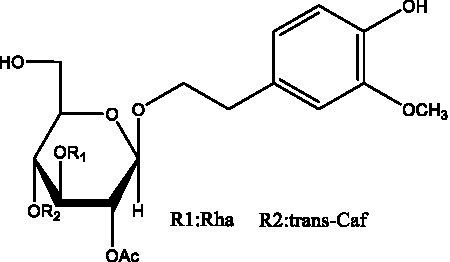	Salsasides E	(42)	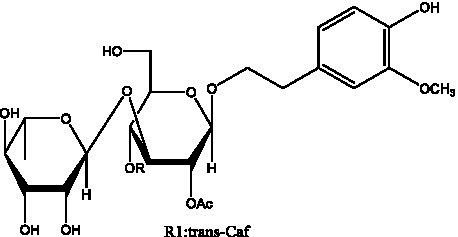	Salsasides F	(42)
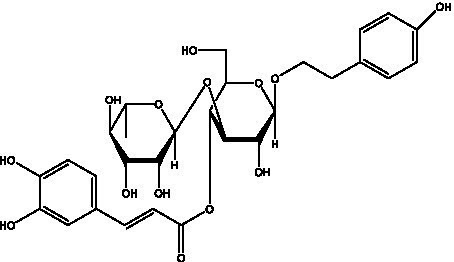	Syringalide A 3′- α-L-rhamnopyranoside	(43)	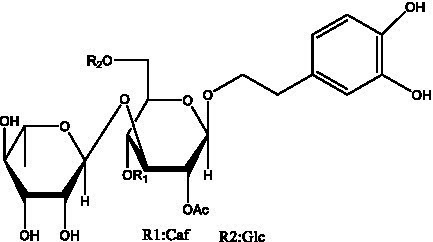	Tubuloside A	(32)
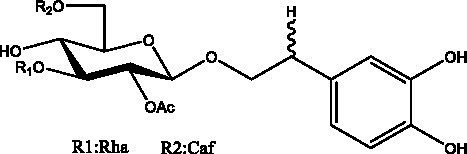	Tubuloside B	(32)	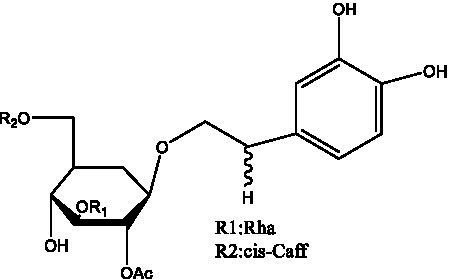	Cis-Tubuloside B	(12)
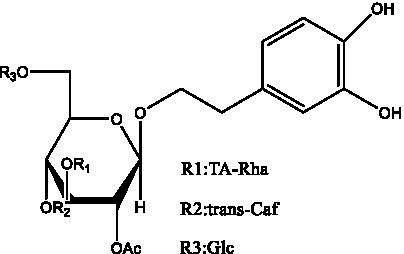	Tubuloside C	(11, 27)	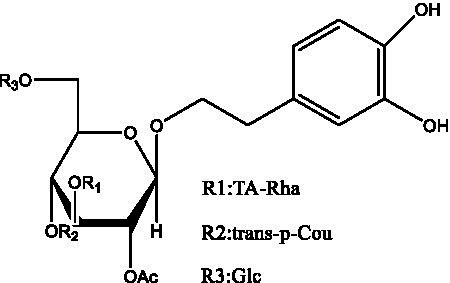	Tubuloside D	(11, 27)
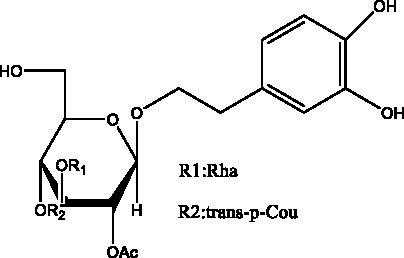	Tubuloside E	(11, 27)	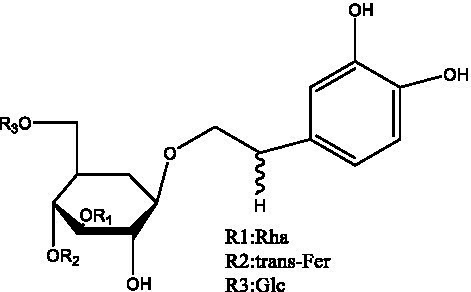	Wiedemanninoside C	(12, 27)
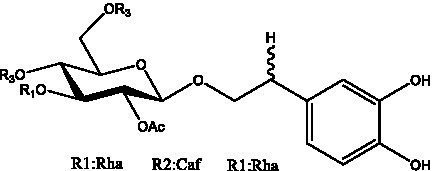	2′-O-acetylpoliumoside	(40)	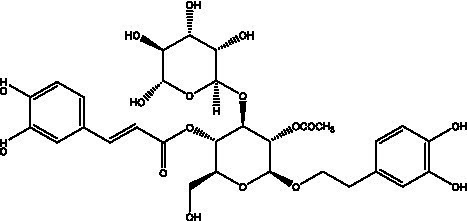	2’-Acetylacteoside	(26, 30)
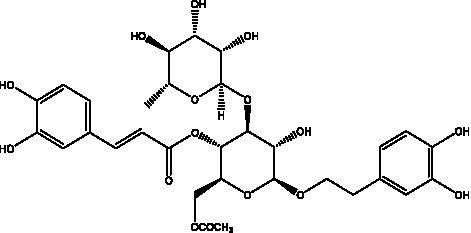	6’-Acetylacteoside	(26)	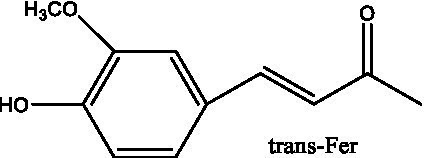	trans-Fer	
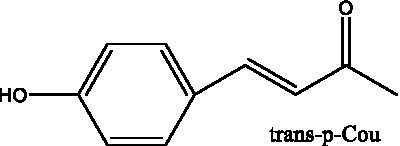	trans-p-Cou		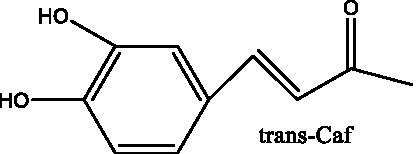	trans-Caf	

Glc: β-glucopyranose. Rha: α-L-rhamnopyranose; Ac: acetyl; Cd: C. deserticola; Ct: C. tubulosa; Csa: C. salsa; Csi: C. sinensis; Cp: C. phelypaea.

#### 2.2.2. Iridoids

Iridoid is one of the main chemical constituents of cistanche and has antibacterial, anti-inflammatory and analgesic effects (44). Jianghua et al. (45) used column chromatography techniques such as macroporous resin and activated carbon to separate cycloen ether mushroom compounds from purified salt cistanche and successfully isolated two compounds. Li et al. (18) found 2 iridoids by using LC/QTOF MS/MS. Wenjing et al. (46) used the HPLC-IT-TOF-MS method to qualitatively analyze the chemical composition of desert cistanche flowers and lignified stems, and found that there were more cycloen ether terpenoids in the flowers and stems of cistanche than in the fleshy stems. At present, there are 27 kinds of cycloen ether terpenoid active substances isolated from cistanche (Table 3). And the structure of these active substances contain glucose monoglycosides, which provides new ideas for the drug research and development of cistanche, and also promotes the research of cistanche in neuroprotective, hepatoprotective and hypoglycemic blood lipids (52).

**Table 3:** Iridoids from Cistanche species.

Name	Chemical structure	Ref	Name	Chemical structure	Ref
					
Adoxosidic acid	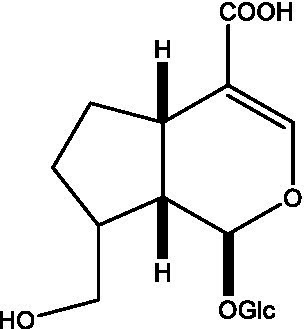	(47)	Antirrhide	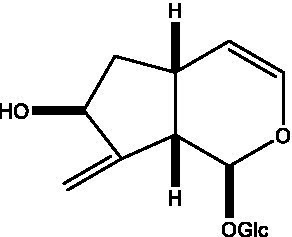	(48)
Ajugol/Leomuride	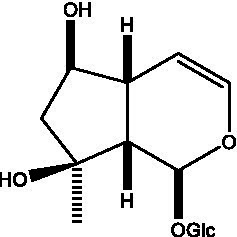	(35, 48)	Argyol	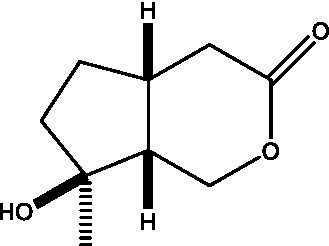	(48)
Bartsioside	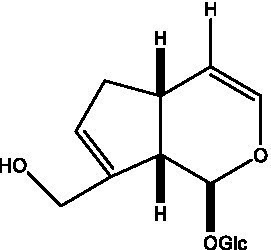	(48, 49)	Catalpol	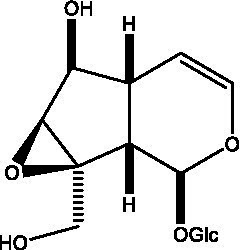	(48)
Cistanin	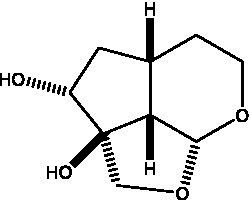	(50)	Cistachlorin	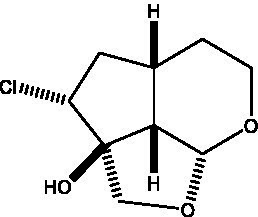	(50)
Cistadesertoside A	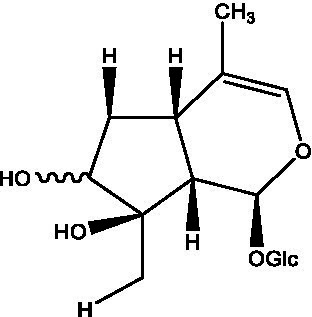	(51)	Geniposide	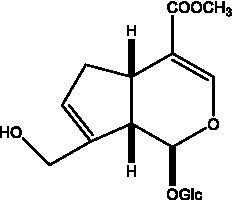	(35)
Geniposidic acid	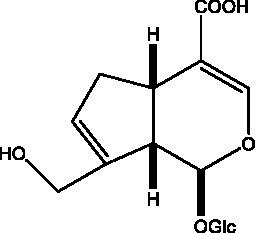	(48, 49)	Gluroside	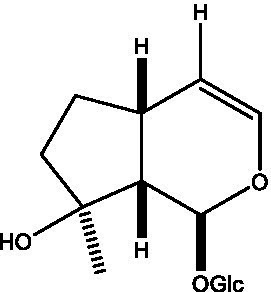	(30, 48, 49)
Kankanol	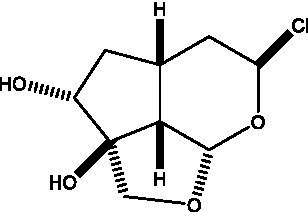	(48)	Kankanoside A	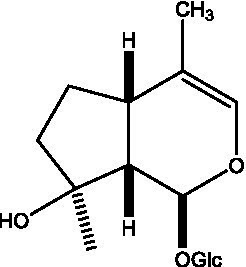	(48)
Kankanoside B	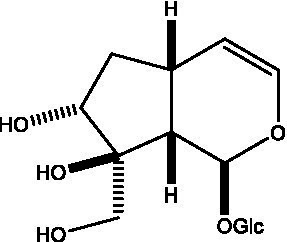	(48)	Kankanoside C	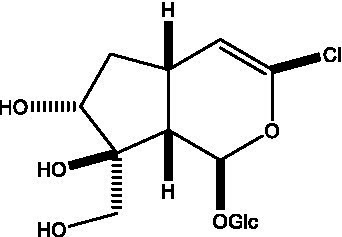	(48)
Kankanoside D	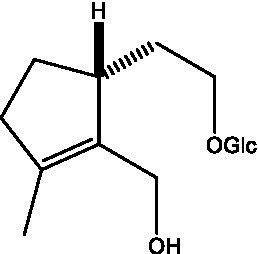	(48)	Kankanoside L	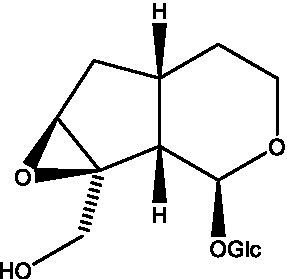	(27)
Kankanoside M	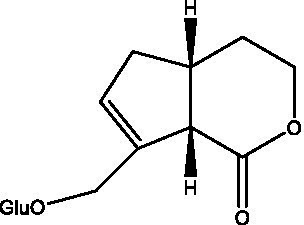	(27)	Kankanoside N	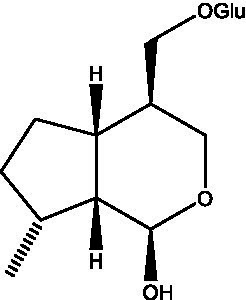	(27)
Mussaenosidic acid	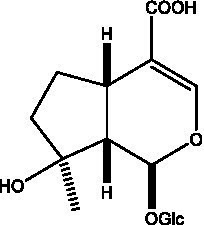	(44, 50)	Mussaenoside	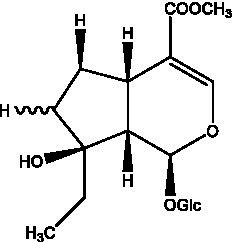	(35)
Phelypaeside	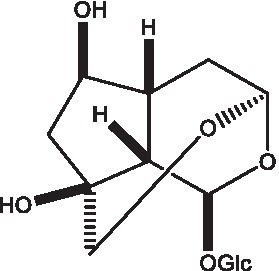	(11)	6-Deoxycatalpol	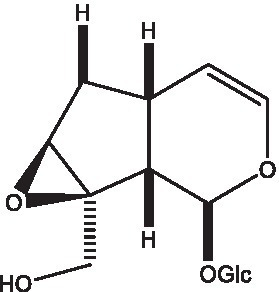	(50)
8-Epideoxyloganic acid	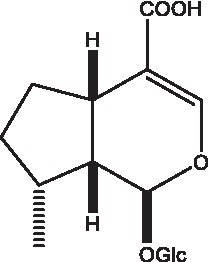	(34, 49)	8-Epiloganic acid	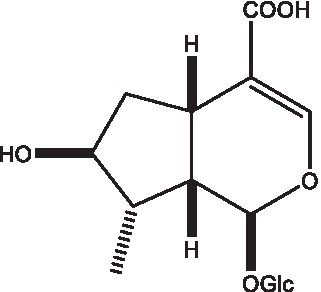	(35)
8-Epiloganin	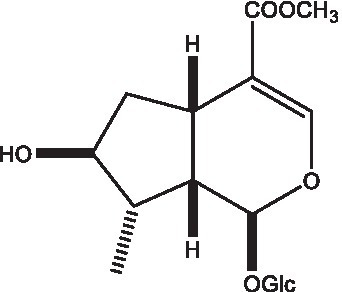	(35)			

Glc: β-glucopyranose. Rha: α-L-rhamnopyranose; Ac: acetyl.

#### 2.2.3. Lignans

Lignan is a large class of compounds containing two phenyl propane units (53), basically present in plants, is a plant hormone, which has been found to have biological properties such as scavenging free radicals, antioxidants, and antivirals in the body (54). Zedong et al. (55) conducted a chemical composition study of desert cistanche by using chromatographic analysis techniques, and isolated 11 lignan compounds, providing new discoveries for the study of lignans in cistanche. At present, there are 17 lignan compounds isolated from cistanche (Table 4), of which syringin, syringaresinol O-β-D-glucopyranoside and pineoresinol are the earliest discovery, which greatly promotes scientists’ research on the estrogen effects of cistanche and provides an alternative for finding alternative synthetic estrogen research.

**Table 4:** Lignans from Cistanche species.

Compounds name	Chemical structure	Ref	Compounds name	Chemical structure	Ref
Alaschanioside A	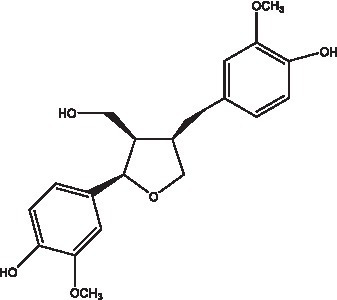	(55)	Citrusin A	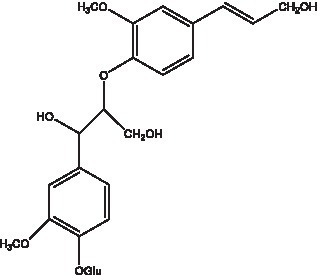	(55)
Conicaoside	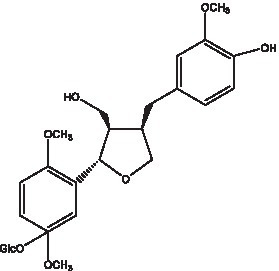	(55)	Ddehydrodiconiferyl alcohol 4-O-β-D-glucopyranoside	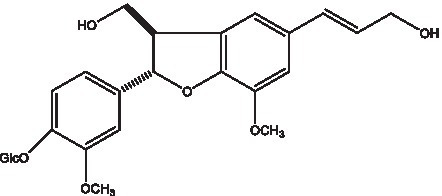	(55)
Dehydrodiconiferyl alcohol γ’-O-β-D-glucopyranoside	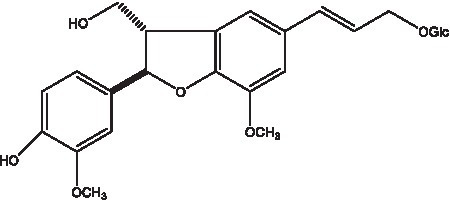	(34, 55)	Eucommin A	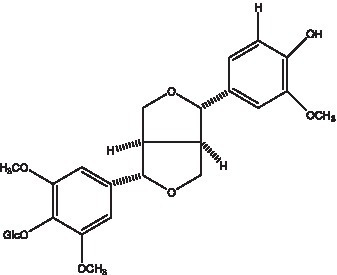	(55)
Isoeucommin A	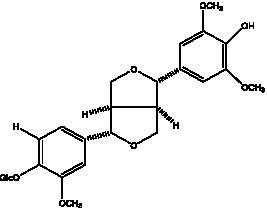	(55)	Isolariciresinol-9′-O-β-D-glucopyranoside	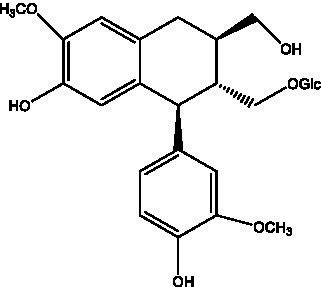	(48)
Lariciresinol 4-O-β-D-glucopyranoside	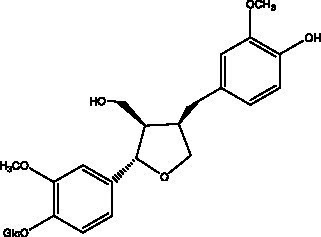	(55)	Lariciresinol 4’-O-β-D-glucopyranoside	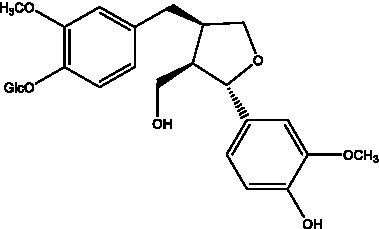	(55)
Liriodendrin	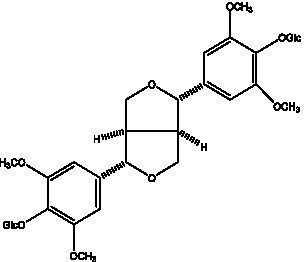	(55)	Lariciresinol 4′-O-β-D-glucopyranoside	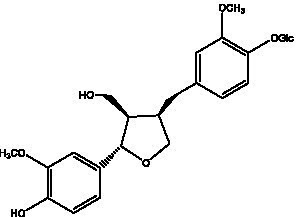	(55)
(+)-Pinoresinol	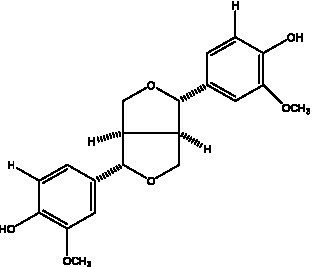	(56)	(+)-Pinoresinol O-β-D-glucopyranoside	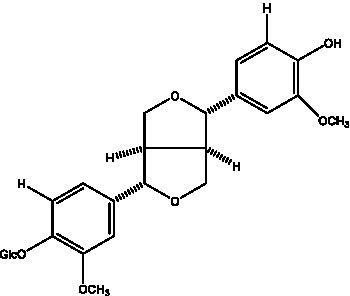	(55)
(+)-syringaresinol	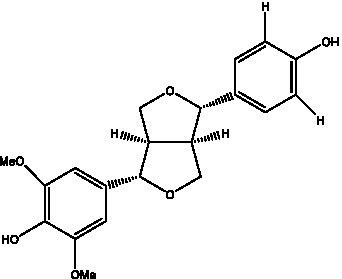	(12)	(+)-Syringaresinol O-β-D-glucopyranoside	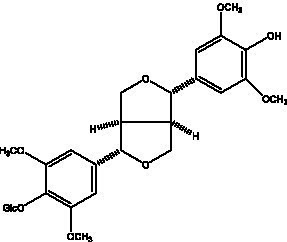	(56)
Syringin	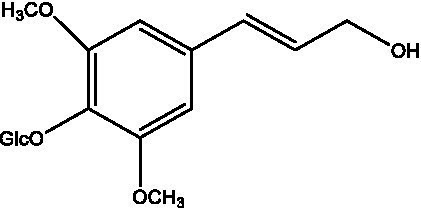	(56)			

#### 2.2.4. Polysaccharides

Polysaccharides are widely present in the composition of animals, plants and microbial cells (57, 58). And polysaccharides can be used as a substance for storing energy in organisms, playing a role in immunomodulation, antiviral, anti-cancer, hypoglycemic, prevention and treatment of heart disease and laxative (59, 60). At present, the research on cistanche polysaccharides is mostly isolated and purified, and the research on the pharmacological effects of polysaccharides has gradually begun to deepen. Xing-hui et al. (61) took Xinjiang desert cistanche as raw material, after process optimization and extraction. It is finally concluded that the use of water immersion extraction method at 75°C, the material-to-liquid ratio is 1:55, the extraction rate of 165 min polysaccharide extraction is the highest, and combined with papain to remove protein, macroporous resin decolorization has the highest polysaccharide recovery rate and the best purification effect. Table 5 is one of the 11 species of cistanche polysaccharides that have been studied so far, and most cistanche polysaccharides are composed of glucose, galactose, rhamnose, arabinose, and fructose. The study of cistanche polysaccharides provided a basic basis for understanding its structure, composition and chemical properties, and also has a huge impact on its biological activity research.

**Table 5:** Polysaccharides from Cistanche species.

Name	Composition	Ref
ACDP-2	1,4-D-gal and D-glu, containing predominantly a branching point at the C6	(62)
CDA-0.05	contained 1, 4-linked α-D-Glcp, 1, 4, 6-linked α-D-Glcp and 1, 4-linked β-D-Galp, with branches of T-linked α-D-Glcp attached at C-6 of 1, 4, 6-linked α-D-Glcp residues	(63)
CDA-1A	an α-(1 → 4)-D-glucan with α-(1 → 6)-linked branches attached to the O-6 of branch points	(64)
CDA-3B	an RG-I polysaccharide containing a typical rhamnogalacturonan backbone and arabinogalactan or arabinan branches	(64)
CLP-1	mannose 2.78%, galacturonic acid 4.07%, glucose 88.62%, galactose 1.80% and arabinose 2.39%	(65)
CLP2	rhamnose 5.79%, galacturonic acid 7.78%, glucose 9.99% and galactose 13.30%.	(65)
CTP	rhamnose, mannose, glucose, and galactose	(66)
CDP-4	straight-chain glucose	(66)
SPA	glucose and galactose are mainly composed of arabinose, rhamnose and mannose	(67)
-	α-l,4-D-glucan, α-L-arabino-3,6-β-D-galactan, pectic polysaccharides and 4-O-methyl-D-glucurono-D-xylan.	(68)
-	glucose, galactose, rhamnose, arabinose and fructose	(69)

#### 2.2.5. Other compounds

**Figure 1:**
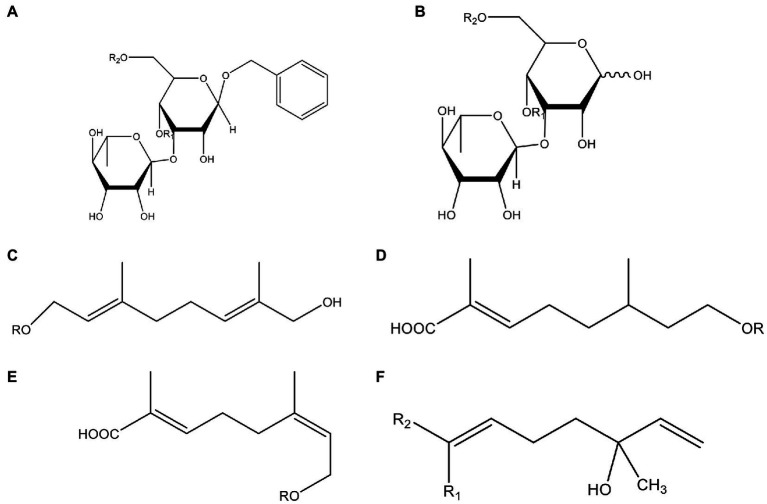
Some chemical structures in cistanche.

In addition, cistanche also contains many other chemical components, Li et al. (18) used LC/QTOF-MS/MS to identify and analyze 1 monoterpenoids. Qing-Qing et al. (12) used mass spectrometry analysis method, but also isolated benzyl alcohol glycosides, phenylacryl oligosaccharides, monoterpenoids and nitrogen-containing compounds, etc., of which the chemical structure of benzool glycoside compounds and phenylacryl oligosaccharides has similarities with the structure of phenylethanol glycoside compounds, and contains glucose in the structure. These compounds are described in Table 6 (Figure 1) and provide a reference for quality control of cistanche by analyzing these chemical components.

**Table 6:** Other compounds from Cistanche species.

Compound category	Name	R	Ref
Benzyl alcohol glycosides	salsaside A (Figure 1A)	R_1_ = H R_2_ = trans-Caf	(42)
	salsaside B (Figure 1A)	R_1_ = trans-Caf R_2_ = H	(42)
	salsaside C1 (Figure 1A)	R_1_ = p-trans-Cou R_2_ = H	(42)
	salsaside C2 (Figure 1A)	R_1_ = p-cis-Cou R_2_ = H	(42)
Phenylacylated oligosugars	Cistanoside F (Figure 1B)	R_1_ = trans-Caf R_2_ = H	(70)
	Cistanoside I (Figure 1B)	R_1_ = p-trans-Cou R_2_ = H	(27)
	Cistantubulose A1/A2 (Figure 1B)	R_1_ = trans-Caf R_2_ = Glc	(31)
	Cistansinensose A1/A2 (Figure 1B)	R_1_ = trans-Caf R_2_ = Rha	(40)
Monoterpenoids	8-hydroxygeraniol (Figure 1C)	R = H	(47)
	8-hydroxygeraniol-1-β-D-glucopyranoside (Figure 1C)	R = Glc	(71)
	(2E,6R)-8-hydroxy-2,6-dimethyl-2-octenotic acid (Figure 1D)	R = H	(72)
	Kankanoside E (Figure 1D)	R = Glc	(48)
	(2E,6Z)-8-O-β-D-glucopyranoside-2,6-dimethyhyl-2,6-octadienoic acid (Figure 1E)	R = Glc	(48)
	(2E)-2,6-dimethyl-2,7-octadiene-1,6-diol (Figure 1F)	R_1_ = CH_3_ R_2_ = CH_2_OH	(73)
	(2Z)-2,6-dimethyl-2,7-octadiene-1,6-diol (Figure 1F)	R_1_ = CH_2_OH R_2_ = CH_3_	(73)
Nitrogen-containing	Uridine	-	(45)
	Inosine	-	(27)
	2’-O-methyladenosine	-	(27)
	(3R)-3-hydroxy-1-methyl-2-pyrrolidinone	-	(45)
	(3R)-3-hydroxy-2-pyrrolidinone		(45)
	(2,5-dioxo-4-imidazolidinyl)-carbamic acid	-	(74)
	Succinimide	-	(29)
	2-methanol-5-hydroxy-pyridine	-	(73)
	betaine	-	(75)

Caf: caffeoyl; Cou: coumaroyl; Glc: β-D-glucopyranosyl; Rha: α-L-rhamnopyranosyl.

## 3. Health benefits of cistanche consumption

**Figure 2:**
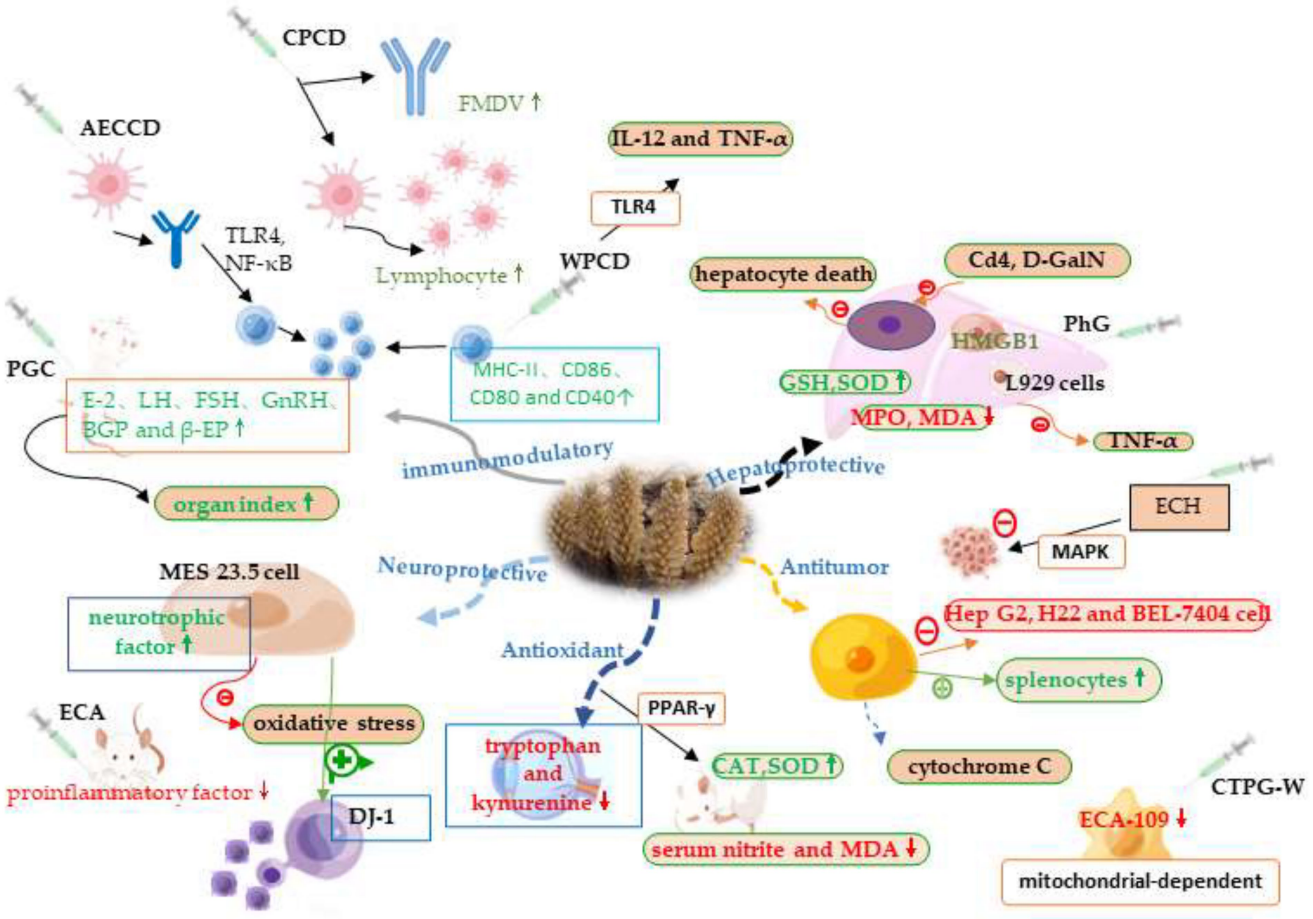
Immunomodulatory effect mechanisms of cistanche.

Cistanche contains a variety of biologically active ingredients. It has different pharmacological mechanisms of action in the body, has been studied that cistanche not only plays an important role in lowering lipids and hypoglycemicity, preventing osteoporosis, but also plays an active role in antioxidant, liver protection, anti-cancer, immunomodulation, Figure 2 briefly summarizes some of the mechanisms of action of cistanche. It can be seen that eating cistanche has many benefits for health.

### 3.1. Immunomodulatory effects

Long-term studied have shown that cistanche polysaccharide is the basis for cistanche to play an immunomodulatory role, which can play a role in regulating the body’s immunity by promoting lymphocyte proliferation and enhancing phagocyte activity. Li et al. (76) evaluated the regulatory activity of crude polysaccharides of Cistanche deserticola (CPCD) on activating dendritic cells (DCs). And the final results showed that CPCD increased the specific antibody of foot-and-mouth disease vaccine (FMDV), promoted lymphocyte proliferation, and concluded that cistanche polysaccharide has a regulatory effect on cellular immunity. Feng et al. (77) extracted water-soluble polysaccharides from cistanche, studied the immune efficacy and efficacy of aqueous extracts of cultivated *Cistanche deserticola Y.C. Ma*(AECCD) through *in vitro* experiments *in vitro*. And the final test results showed that AECCD can activate a long-lasting and effective antigen-specific immune response through DC, and can also participate in regulating cytokine expression through TLR4-related NF-κB pathway, which can promote related cell proliferation, activate T cell response, and is a potential immunomodulator. Tian et al. (78) selected Wistar female mice, except for the blank group, the rest of the rats were excised the left ovary, 80% of the right ovary was excised, administered by gastric lavage, phenylethanoid glycosides of cistanche herb (PGC) high, medium, low suspension which is 450 mg/(kg day), 133.33 mg/(kg day), 66.67 mg/(kg day), 33.33 mg/(kg day), the blank group was fed the same volume of distilled water for 30 consecutive days. After the last administration, blood was taken from the abdominal aorta and the levels of E-2, LH, FSH, GnRH, BGP and β-EP in serum were determined, respectively, and the final results showed that PGC increased the activity, the organ index (thymus, spleen, uterus), E2, T, BGP level in serum, β-EP level in plasma, AR level in hypothalamus, ER level in hypothalamus, pituitary. Zhang et al. (79) used water-extractable polysaccharides of CD(WPCD) to study, they found WPCD significantly promoted the maturation and function of murine marrow-derived dendritic cells (BM-DCs) through up-regulating the expression levels of MHC-II, CD86, CD80, and CD40, allogenic T cell proliferation, and the yields of IL-12 and TNF-α *via* toll-like receptor4 (TLR4). So WPCD activates DCs through the TLR4 signaling pathway, triggering humoral and cellular immunity. Therefore, cistanche polysaccharides and PhG play an important role in immune regulation (Table 7).

**Table 7:** Immunomodulatory mechanism of cistanche.

Functional ingredients	Cell line/Animal model or method	Occurring mechanism or effect	Evaluation of research findings	Ref
CPCD	Modulator activities of CPCD on activating dendritic cells (DCs) and the adjuvant potential for foot and mouth disease vaccine (FMDV)	TLR-2 or TLR-4 antibodies suppressed levels of CPCD-mediated CD40 and CD86 as well as IL-6 and IL-1β in DCs. CPCD induced the phosphorylation of MAPKs related molecules and NF-κB.	CPCD could effectively stimulate stronger humoral and cellular responses by modulating DC activation through TLR-2/TLR-4 related MAPKs and NF-κB pathway.	(76)
AECCD(aqueous extracts of cultivated Cistanche deserticola)	ICR mice against ovalbumin (OVA), dendritic cells (DC) activation mechanism by AECCD	AECCD elicited vigorous and long-term IgG responses with mixed Th1/Th2 responses and up-regulated levels of Th-associated cytokines (CD4 + IL-4, CD4 + IFN-γ and CD8 + IFN-γ).	AECCD could elicit potent and durable antigen specific im mune responses through DC activation.	(77)
PGC	Wistar female rats were selected. The left ovaries for all rats except in the blank control group(BC) were removed, and the right ovaries were removed in 80%.	PGC increase the activity, the organ index (thymus, spleen, uterus), E-2, T, BGP level in serum, beta-EP level in plasma, AR level in hypothalamus, ER level in hypothalamus, pituitary, uterus in perimenopausal model rats. And it also reduced FSH, LH, GnRH level in serum, and improved uterine and ovarian lesions in perimenopausal model rats.	Each dose of PCG could counteract the disorder of sex hormone in perimenopausal model rats, correct the imbalance of ER and AR level, enhance and restore the effect of uterus and the nerve cells of hypothalamic, and improve immune function.	(78)
WPCD	DCs from C57BL/6 mice, ovalbumin (OVA) (Sigma) was used as the model antigen and female ICR mice	WPCD significantly promoted the maturation and function of murine marrow-derived dendritic cells (BM-DCs) through up-regulating the expression levels of MHC-II, CD86, CD80, and CD40, allogenic T cell proliferation, and the yields of IL-12 and TNF-α *via* toll-like receptor4 (TLR4), as indicated by *in vitro* experiments.	WPCD could modulate immune responses *in vitro* and *in vivo*.	(79)

### 3.2. Neuroprotective effect

Parkinson’s disease is a chronic disease characterized by dysfunction of the central nervous system, from Table 8, it can be seen that cistanche has a protective effect on the nerves. By gavaging Wistar rats Herb Epimedii (Epimedium), Semen Cuscutae (Dodder Seed), or Herb Cistanches (Desertliving Cistanche), then the sera of different groups of mice were compared and analyzed by enzyme-linked immunosorbent assay and MES23.5 cells in the logarithmic phase were cultured in medium with 15% drug-containing serum added for 24 h. The results showed that serum containin Cistanches increased the expression of nerve growth factor, brain-derived neurotrophic factor, and glial cell line-derived neurotrophic factor in injured MES23.5 cells, so *Cistanche deserticola* can protect nerve cells by regulating the expression of apoptosis related factors and neurotrophic factors in MES23.5 cells (80). Echinacoside (ECA) is one of the main compounds of Cistanche deserticola. By intraperitoneal injection of echinacoside into rats, the results showed that the effect of pretreatment with echinacoside on the expression of proinflammatory factor genes in the hippocampus of kainic acid-induced epileptic rats would be weakened. Therefore, echinacoside is the potentially useful in the prevention of epilepsy (81). Ischemic stroke is a disease with high morbidity and mortality, cistanche deserticola polysaccharides (CDP) has always been considered to have neuroprotective effects, through cell experiments have found that CDP can inhibit oxidative stress and regulate the secretion and expression of DJ-1 has a protective effect on nerve damage induced by OGD/RP. So it has clinical significance for the treatment of ischemic stroke diseases (82). The Parkinson’s disease like model induced by the neurotoxin 1-methyl-4-phenyl-1,2,3,6-tetrahydropyridine(MPTP) is mainly based on the lack of its ability to produce dopamine in the striatum, resulting in the loss of dopaminergic neurons in the dense part of the substantia nigra, the study found that the mouse model pretreated with PhGs had obvious neuroprotective effect on dopaminergic neurons in substantia nigra by immunohistochemical analysis. Therefore, it is believed that PhG is conducive to the treatment of Parkinson’s disease and plays a neuroprotective role in the human body (83).

**Table 8:** Neuroprotective effect mechanism of cistanche.

Functional ingredients	Cell line/Animal model or method	Occurring mechanism or effect	Evaluation of research findings	Ref
Herba Cistanches	Induced oxidative damage in MES23.5 cells using H2O2	All drug-containing serums improved the survival rate of H2O2-injured MES23.5 cells, inhibited pro-apoptotic FasL and caspase-3 expression, promoted anti-apoptotic Bcl-2 expression.	Chinese medicines used to tonify the kidney can protect nerve cells by regulating the expression of apoptosis-related factors and neurotrophic factors in MES23.5 cells.	(80)
ECA	Kainic acid-induced seizures in rats	Rats pre injected with Echinacea could reduce the effect of kainic acid on glutamate concentration in mice, slow down neuron loss and microglia activation, and inhibit the expression of proinflammatory cytokine genes in hippocampus	Echinacoside is the potentially useful in the prevention of epilepsy	(81)
CDP	PC12 cell model	CDP (0.05, 0.5 and 5 mu g/ml) attenuated PC12 cell death, preserved MMP and calcium homeostasis; inhibited oxidative stress and decreased cell apoptosis. Moreover, CDP (5 mu g/ml) markedly stimulated DJ-1 secretion and expression.	CDP exerts neuroprotective effect against OGD/RP-induced injury by inhibiting oxidative stress and regulating the DJ-1 pathway	(82)
PhG	Parkinson’s mouse model induced by neurotoxin 1-methyl-4-phenyl-1,2,3,6-tetrahydropyridine (MPTP)	Neuroprotective effects of PhGs on nigral dopaminergic neurons were confirmed by the results of immunohistochemical staining.	PhG has neuroprotection	(83)

### 3.3. Antioxidant effect

Among the existing health foods of *Cistanche deserticola* in the market, many products claim to have antioxidant effect (Table 9). In the research on the oxidation of *Cistanche deserticola*, Zhang et al. (86) compared and analyzed the chemical components and biological effects of the raw product of Cistanche deserticola slices (RCD) and its wine steam processed product (WSCD). The final results showed that both of them could restore the sex hormone level of model mice and improve the antioxidant effect. By comparing and analyzing the therapeutic effects of antioxidants and cistanche powder on cataracts with senscence accelerated OXYS rats as the test object. The experimental results showed that the antioxidant effect was related to the oxidation rate and level of tryptophan and kynurenine in the lens, and cistanche powder, like antioxidants, helped to slow down the oxidation rate of tryptophan and kynurenine, reduce the level, which can effectively reduce the oxidative stress in the lens and help cataract treatment (84). Some researchers also took rats with senile stress disorder as the research object, through the analysis of serum nitrite and MDA concentration, SOD and CAT activity, Cistanche can reduce nitrite and MDA concentration and increase SOD and CAT activity, so as to reduce the level of oxidative stress (85). Combined with the application of PPAR-γ anti-caking agents, the results show that cistanche can exert antioxidant effects on the development of sevoflurane-induced cognitive dysfunction by activating PPAR-γ signaling. (87) extracted PhG from cistanche, studied the cognitive protection mechanism of PhG pair (SAMP3) model mice, and by measuring MDA concentration, SOD and GSH-Px activity, the results showed that PhG has antioxidant effects. Therefore, cistanche has a positive role in antioxidant research, and studying antioxidant mechanisms helps to provide clinical guidance and recommendations for disease treatment.

**Table 9:** Antioxidant effect mechanism of cistanche.

Functional ingredients	Cell line/animal model or method	Occurring mechanism or effect	Evaluation of research findings	Ref
*Cistanche deserticola*	Senescence accelerated OXYS rats	The oxidation rate of tryptophan and kynurenine in mice supplemented with *Cistanche deserticola* slowed down	*Cistanche deserticola* can slow down the development of cataract	(84)
Cistanche	Sevoflurane-induced aged cognitive dysfunction rat model	*Cistanche deserticola* can reduce oxidative stress by reducing nitrite and MDA and increasing SOD and CAT activities at the same time.	*Cistanche deserticola* can activate PPAR-γ Signal transduction plays an antioxidant role in the development of sevoflurane induced cognitive dysfunction	(85)
PhG	Kidney yang deficiency model	The contents of SOD and MDA in treatment group were higher than those in other groups, the Level of Hormone (T and E2). were increasing	PhG can restore the level of neutral hormones in the kidney yang deficiency model and improve the antioxidant effect	(86)
PhG	AD senescence accelerated mouse prone 8 (SAMP8) model	PHG significantly increased the density of dendritic spines in hippocampal CA1 region, accompanied by increased expression levels of synaptophysin (SYN) and postsynaptic density 95 (PSD-95), decreased MDA content, and increased SOD and GSH PX activities	The ability of PhG to ameliorate cognitive deficits in SAMP8 mice may be related to promotion in synaptic plasticity involving antioxidant processes	(87)

### 3.4. Antitumor effect

Modern pharmacological studies have shown that Cistanche tubulosa phenylethanoid glycosides (CTPG) has anti-tumor effects on a variety of tumor cells (Table 10). At present, through cell experiments and H22 Tumor mouse model studies, CTPG can inhibit the normal growth of Hep G2 and BEL-7404 cells by inducing cell cycle arrest and apoptosis. And *in vivo* test results show that CTPG enhanced the proliferation of splenocytes and reduced the apoptosis of splenocytes induced by cisplatin. It can be seen that CTPG has the effect of inhibiting the growth of tumor cells (88). Yuan et al. (89) studied the anti-tumor mechanism and effect of CTPG on H22 liver cancer cells through a combination of *in vivo* and *in vitro* experiments. The final results showed that CTPG could inhibit the growth of H22 cells, promote the release of cytochrome C, and significantly enhance the signaling pathway. The level of caspase-8 and caspase-9 cleavage, which activates caspase-7 and-3 to cleave PARP, so CTPG can improve the survival rate of liver cancer mice. Fu et al. (90) studied the effect of water-soluble phenylethanoid glycosides of C. tubulosa (CTPG-W) on ECA-109 cell, and the results showed that CTPG-W can significantly reduce cell viability, induce apoptosis of ECA-109 cells through mitochondrial-dependent pathways, and have a high-quality therapeutic effect on esophageal cancer. Echinacoside (ECH), one of the phenylethanoids, isolated from the stems of Cistanches salsa, has been shown to inhibit the proliferation of pancreatic cancer cells by promoting apoptosis, and revealed that ECH inhibits tumor cell growth by regulating MAPK activity (91). So, it can be seen that cistanche has development potential in cancer treatment.

**Table 10:** Antitumor effect mechanism of cistanche.

Functional ingredients	Cell line/Animal model or method	Occurring mechanism or effect	Evaluation of research findings	Ref
CTPG	HepG2 and BEL-7404 hepatocellular carcinoma (HCC) cells, H22 tumor mouse model	CTPG significantly inhibited the growth of HepG2 and BEL-7404 cells through the induction of cell cycle arrest and apoptosis, which was associated with the activation of MAPK pathways characterized by the up-regulated phosphorylation of p38, JNK, and ERK1/2 and mitochondria-dependent pathway characterized by the reduction of mitochondrial membrane potential. The release of cytochrome c and the cleavage of caspase-3, −7, −9, and PARP were subsequently increased by CTPG treatment. CTPG combined with cisplatin further inhibited the growth of H22 cells and reduced the side effects of cisplatin.	CTPG inhibited the growth of HCC through direct antitumor effect and indirect immunoenhancement effect, and improved the antitumor efficacy of cisplatin	(88)
CTPG	H22 cells; tumor mouse model established using male Kunming mice	CTPG treatment significantly suppressed H22 cell growth in a dose and time dependent manner; significantly increased Bax/ Bcl-2 ratio, reduced Δψm and enhanced the release of cytochrome c; the levels of cleaved caspase-8 and caspase-9 in both extrinsic and intrinsic signaling pathways were significantly increased that sequentially activated caspase-7 and-3 to cleave PARP.	CTPG suppressed H22 cell growth through both extrinsic and intrinsic apoptosis pathways	(89)
CTPG-W	Eca-109 cells	CTPG-W significantly reduced the viability of Eca-109 cells through the induction of apoptosis and cell cycle arres; and the levels of cytochrome c and c-Jun NH2-terminal kinase were increased, which upregulated the levels of cleaved-poly (ADP-ribose) polymerase and cleaved-caspase-3, −7 and -9, but not caspase-8.	CTPG-W induced apoptosis of Eca-109 cells through a mitochondrial-dependent pathway	(90)
ECH	SW1990 pancreatic adeno- carcinoma cells	ECH can markedly inhibit the proliferation of pancreatic adenocarcinoma cells by inducing the production of reactive oxygen species and the perturbation of mitochondrial membrane potential and thus triggering apoptosis; and ECH represses tumor cell growth through modulating MAPK activity	ECH inhibits cancer development	(91)

### 3.5. Hepatoprotective effect

Liver damage, cirrhosis and fatty liver disease are currently more common liver diseases, affecting people’s health (Table 11). Morikawa et al. (92) used the methanolic extract from fresh stems of Cistanche tubulosa to study the protective effect of PhG on the liver, they used a model of liver damage caused by D-galactosamine (D-Galn)/lipopolysaccharide (LPS) and found that PhG can inhibit hepatocyte death, reducing the toxic effect of TNF-a on L929 cells. It is believed that PhG has a hepatoprotective effect *in vivo*. Xiong et al. (93) used NADPH/CCI4-induced fatty liver rat model, observing the inhibitory effect of PhGs compounds on hepatocytes efficiently, they prevented cell damage induced by exposure to Cd4 or o-galactosamine(D-GalN), thus it was proved that PhGs have a protective effect on liver cells. Cui et al. (94) used the GalN/LPS-induced-acute-hepatic-injury mouse model, found that the plasma ALT and AST levels of mice given to the ACT group were significantly reduced. The content of GSH and SOD was increased in the metabolites of liver tissue, the level of MPO and MDA was reduced, and the pathological changes of liver histopathology were effectively improved. And the inflammatory cytokine HMGB1 in serum was effectively improved. TNF-α and IL-6 levels have a regulatory effect, which suggests that ACT can effectively prevent liver damage. Because SOD is a typical antioxidant enzyme, GST is a soluble protein located in cytoplasm that plays an important role in liver detoxification. So SOD and GST are considered as indicators of alcoholic hepatotoxicity, and liver MDA and TG can also be used as indicators of hepatotoxicity. In the end, alcohol has been found to significantly reduce SOD and GST levels and elevate MDA and TG levels in mice, while CDP-C mitigates this change, and histopathological observations of liver slices suggest that CDP-C can significantly restore alcohol damage to liver tissue (95). Therefore, it is believed that cistanche products play a positive role in liver protection and liver protection.

**Table 11:** Hepatoprotective effect mechanism of cistanche.

Functional ingredients	Cell line/Animal model or method	Occurring mechanism or effect	Evaluation of research findings	Ref
PhG	D-galactosamine (D-GalN)/lipopolysaccharide (LPS)-induced liver injury in mice	20-acetylacteoside and tubuloside A inhibited D-GalN-induced death of hepatocytes, isolates and cistantubuloside B1 also reduced TNF-a-induced cytotoxicity in L929 cells	Fresh cistanche extract has a protective effect on the liver	(92)
PhGs	NADPH/CCI4-induced lipid peroxidation in rat liver microsomes	The tested four phenylethanoids consecutively inhibited both hepatocytes lipid peroxidation and AST release to the medium and alleviated the cell death induced by CCI4	Phenylethanoids were potent hepatoprotective agents against CCI4 intoxication.	(93)
Acteoside (ACT)	GalN/LPS-induced-acute-hepatic-injury mouse model	GalN/LPS administration markedly increased MPO levels in liver tissues, indicating significant macrophage infiltration, whereas the CA, HT, and 3-HPP pretreatments reduced the levels of MPO; the ACT group reduced levels of HMGB1, TNF-α, and IL-6	ACT metabolites could be responsible for the potent hepatoprotective activity as well as the other therapeutic effects.	(94)
CDP-C	Hep G2 and Er Guo-tou white spirit was used to establish liver injury model in ICR mice	*In vitro* research, CDP-C promoted viability of HepG2 cells; CDP-C can reduce the contents of MDA and TG in liver, and modulate the enzyme activities	CDP-C can be used in the treatment of alcoholic liver disease	(95)

### 3.6. Other pharmacological effects

In addition, cistanche has pharmacological effects such as immunomodulatory, antioxidant, anti-cancer and hepatoprotective liver protection. It also plays an active role in preventing osteoporosis, maintaining the health of the intestinal flora, and protecting heart health. By observing the effect of cistanche on the content and functional parameters of myocardial mitochondrial glutathione content and functional parameters in rats. It was found that cistanche alcohol extracts could increase mitochondrial glutathione content, reduce mitochondrial Ca^2+^ content, increase mitochondrial membrane potential, induce the production of unconjugated proteins. Thereby enhancing the cardiac mitochondrial glutathione status and respiration in rats, and playing a cardioprotective role (96). By giving rats with excision of ovaries, cistanche extract was found to significantly increase bone density (BMD), bone mineral content (BMC), maximum load, maximum load displacement, maximum load stress, self-breaking load, self-breaking displacement, self-breaking stress, and can significantly improve blood antioxidant enzyme activity, reduce blood calcium, zinc, copper levels. Thereby proving that cistanche has the effect of preventing osteoporosis (97). Fu et al. (98) fed rats cistanche polysaccharides, through pharmacokinetic parameters and intestinal microbial composition studies found that cistanche polysaccharides can increase the growth of intestinal beneficial flora, improve the production of short-chain fatty acids, promote the body’s intestinal health. Zhu et al. (99) used diet/streptozotocin (STZ)-induced diabetic rats, the research found that total glycosides of cistatnche tubulosa (TGCT) could improve oral glucose tolerance test (OGTT), area under curve of glucose (AUC-G)and insulin sensitivity, increase glycogen content and glucose metabolism enzyme activity, regulate blood lipid changes. So TGCT can be considered to have a lipid-lowering and glucose lowering effect. As a traditional tonic Chinese herbal medicine, cistanche has a far-reaching influence in regulating the function of the body, and its development as a food has far-reaching research value in nutrition and health research.

## 4. Conclusion

Cistanche is a kind of traditional Chinese medicine with high medicinal value in the history of Chinese medicine, known as “desert ginseng,” modern composition analysis studies have shown that cistanche contains hundreds of active ingredients, of which the content and type of phenethyl glycosides are the most extensively studied. In 2021, the European Food Safety Authority (EFSA) passed (EU) 2015/2283 (100), which means that cistanche water extract PhG has the potential to enter EFSA’s novel food list and it may have the opportunity to be produced in Europe as a food supplement and food for special medical purposes. In conjunction with China, cistanche is listed as a dual-use substance for medicine and food, and is included in the list of food and drug homologous substances. And Liao et al. (101) conducted genotoxicity and 28-day repeated dose toxicity test, it is fully proved that cistanche has a certain degree of edible safety. This review summarized the composition of cistanche, briefly introduce the health benefits of cistanche on the human body, and describes its mechanism of bioactive ingredient. However, due to the short time that cistanche has been included in the list of medicinal and food homologous substances, the current product processing technology is still based on processing drugs, slicing and synergistic, so the development of cistanche foods is limited. And its functional characteristics are limited to its pharmacokinetic research, which is different from the nutrition research of traditional foods. Therefore, we believe that cistanche has great potential for development in functional food applications, as a food and drug homologous substances. In the future, researchers should strengthen its functional characteristics research, further analyze the chemical composition, active ingredient quality control and influence mechanism of cistanche. To establish a standardized composition extraction and processing process and quality determination standards for cistanche. So as to better promote human health.

## Author contributions

SZ: conceptualization, methodology, writing—original draft preparation, and visualization. YZ and SZ: software and formal analysis. DF: validation. DF and HD: investigation. HD: resources. WY: data curation, writing—review and editing, and funding acquisition. WY and SZ: supervision. WY and YJ: project administration. All authors have read and agreed to the published version of the manuscript.

## Funding

This research was funded by Beijing Municipal University Classification Development Project.

## Conflict of interest

YJ was employed by Inner Mongolia Sankou Biotechnology Co., Ltd.

The remaining authors declare that the research was conducted in the absence of any commercial or financial relationships that could be construed as a potential conflict of interest.

## Publisher’s note

All claims expressed in this article are solely those of the authors and do not necessarily represent those of their affiliated organizations, or those of the publisher, the editors and the reviewers. Any product that may be evaluated in this article, or claim that may be made by its manufacturer, is not guaranteed or endorsed by the publisher.
