# Supplementary material for: Analysis of the active ingredients and health applications of cistanche
Source: Front Nutr. 2023 Mar 3;10:1101182. doi: 10.3389/fnut.2023.1101182 (PMC10042234; doi:10.3389/fnut.2023.1101182)
Supplement: Supplementary file 1 [file Data_Sheet_1.docx]

Supplementary Material

# Supplementary Data

**Table 1.** General nutrient and trace element content in cistanche

| **Nutrient** | **Content (per 100g of original fruit)** | **Element** | **Content(μg/g original fruit）** |
| --- | --- | --- | --- |
| moisture(g) | 7.16 | K | 7.60x10^4^ |
| fat(mg) | 12 | Na | 1.14x10^5^ |
| cholesterol(μg) | 102 | Ca | 3.63x10^4^ |
| ash(g) | 0.24 | Fe | 1.05x10^4^ |
| dietary fiber(g) | 0.19 | Mg | 9.09x10^3^ |
| reducing sugar(mg) | 7.34 | Mn | 2.02x10^2^ |
| total acid(g) | 1.56 | Zn | 1.07x10^2^ |
| V_A_(mg) | 2.42 | Cu | 9.42x10^2^ |
| V_C_(mg) | 3.12 | Sr | 1.55x102 |
|  |  | Ni | 11.53 |

**Table 2.** Phenylethanoid glycosides from Cistanche species

| **Chemical Structure** | **PhGs compound** | **Ref** | **Chemical Structure** | **PhGs compound** | **Ref** |
| --- | --- | --- | --- | --- | --- |
|  | acteoside | (26) |  | Cis-acteoside | (27) |
|  | arenarioside | (27) |  | Cistanoside A | (26,28) |
|  | Cistanoside B | (28,29) |  | Cistanoside C | (26,30) |
|  | Cistanoside D | (30) |  | Cistanoside E | (28) |
|  | Cistanoside F | (31,32) |  | Cistanoside G | (32) |
|  | Cistanoside H | (32) |  | Cistanoside J | (33) |
|  | Cis-cistanoside J | (34) |  | Cistanoside K | (33) |
|  | Cis-cistanoside K | (12) |  | Cistanoside L | (33) |
|  | Cistanoside M | (33) |  | Cistanoside N | (33) |
|  | Cistantubuloside A | (31) |  | Cistantubuloside B1 | (31) |
|  | Cistantubuloside B2 | (31) |  | Cistantubuloside C1/C2 | (31) |
|  | Cistansinenside A | (35) |  | Cistansinenside B | (35) |
|  | Campneoside Ⅰ | (36) |  | Campneoside Ⅱ | (36) |
|  | Crenatoside | (27) |  | Decaﬀeoylacteoside | (37) |
|  | Echinacoside (ECH) | (26) |  | Eutigoside A | (38) |
|  | Isoacteoside | (26) |  | IsocampneosideⅠ | (36) |
|  | Isocistanoside C | (39) |  | cis-isocistanoside C | (12) |
|  | Isosyringalide A 3′-α-L-rhamnopyronoside | (39) |  | Jionoside D | (40) |
|  | Kankanoside F | (32,33) |  | Kankanoside G | (32) |
|  | Kankanosides H_1_ | (27) |  | Kankanosides H_2_ | (27) |
|  | Kankanosides I | (27) |  | Kankanosides J1/ J2 | (36) |
|  | Kankanosides K1/ K2 | (36) |  | Osmanthuside B | (41) |
|  | Osmanthuside B6(E) | (12) |  | Osmanthuside B6(Z) | (26) |
|  | Plantainoside C | (34) |  | Poliumoside | (40,41) |
|  | Pheliposide | (11) |  | Phenylethyl-glucopy ranoside | (34) |
|  | Salidroside | (32) |  | Salsasides D | (42) |
|  | Salsasides E | (42) |  | Salsasides F | (42) |
|  | Syringalide A 3’- α-L-rhamnopyranoside | (43) |  | Tubuloside A | (32) |
|  | Tubuloside B | (32) |  | Cis-Tubuloside B | (12) |
|  | Tubuloside C | (11,27) |  | Tubuloside D | (11,27) |
|  | Tubuloside E | (11,27) |  | Wiedemanninoside C | (12,27) |
|  | 2′-O-acetylpoliumoside | (40) |  | 2’-Acetylacteoside | (26,30) |
|  | 6’-Acetylacteoside | (26) |  | trans-Fer |  |
|  | trans-p-Cou |  |  | trans-Caf |  |

Glc: β-glucopyranose. Rha: α-L-rhamnopyranose; Ac: acetyl; Cd: C. deserticola; Ct: C. tubulosa; Csa: C. salsa; Csi: C. sinensis; Cp: C. phelypaea.

**Table 3.** Iridoids from Cistanche species.

| **Name** | **Chemical Structure** | **Ref** | **Name** | **Chemical Structure** | **Ref** |
| --- | --- | --- | --- | --- | --- |
| Adoxosidic acid |  | (47) | Antirrhide |  | (48) |
| Ajugol/Leomuride |  | 35,48) | Argyol |  | (48) |
| Bartsioside |  | (48,49) | Catalpol |  | (48) |
| Cistanin |  | (50) | Cistachlorin |  | (50) |
| Cistadesertoside A |  | (51) | Geniposide |  | (35) |
| Geniposidic acid |  | (48,49) | Gluroside |  | (30,48,49) |
| Kankanol |  | (48) | Kankanoside A |  | (48) |
| Kankanoside B |  | (48) | Kankanoside C |  | (48) |
| Kankanoside D |  | (48) | Kankanoside L |  | (27) |
| Kankanoside M |  | (27) | Kankanoside N |  | (27) |
| Mussaenosidic acid |  | (44,50) | Mussaenoside |  | (35) |
| Phelypaeside |  | (11) | 6-Deoxycatalpol |  | (50) |
| 8- Epideoxyloganic acid |  | (34,49) | 8-Epiloganic acid |  | (35) |
| 8-Epiloganin |  | (35) |  |  |  |

Glc: β-glucopyranose. Rha: α-L-rhamnopyranose; Ac: acetyl.

**Table 4****.** Lignans from Cistanche species.

| **Compounds name** | **Chemical Structure** | **Ref** | **Compounds name** | **Chemical Structure** | **Ref** |
| --- | --- | --- | --- | --- | --- |
| Alaschanioside A |  | (55) | Citrusin A |  | (55) |
| Conicaoside |  | (55) | Ddehydrodiconiferyl alcohol 4-O-β-D-glucopyranoside |  | (55) |
| Dehydrodiconiferyl alcohol γ'-O-β-D- glucopyranoside |  | (34,55) | Eucommin A |  | (55) |
| Isoeucommin A |  | (55) | Isolariciresinol-9′-O-β-D-glucopyranoside |  | (48) |
| Lariciresinol 4-O-β-D-glucopyranoside |  | (55) | Lariciresinol 4’-O-β-D-glucopyranoside |  | (55) |
| Liriodendrin |  | (55) | Lariciresinol 4′-O-β-D-glucopyranoside |  | (55) |
| (+)-Pinoresinol |  | (56) | (+)-Pinoresinol O-β-D-glucopyranoside |  | (55) |
| (+)-syringaresinol |  | (12) | (+)-Syringaresinol O-β-D- glucopyranoside |  | (56) |
| Syringin |  | (56) |  |  |  |

**Table 5.** Polysaccharides from Cistanche species

| **Name** | **Composition** | **Ref** |
| --- | --- | --- |
| ACDP-2 | 1,4-D-gal and D-glu, containing predominantly a branching point at the C6 | (62) |
| CDA-0.05 | contained 1, 4-linked α-D-Glcp, 1, 4, 6-linked α-D-Glcp and 1, 4-linked β-D-Galp, with branches of T-linked α-D-Glcp attached at C-6 of 1, 4, 6-linked α-D-Glcp residues | (63) |
| CDA-1A | an α-(1→4)-D-glucan with α-(1→6)-linked branches attached to the O-6 of branch points | (64) |
| CDA-3B | an RG-I polysaccharide containing a typical rhamnogalacturonan backbone and arabinogalactan or arabinan branches | (64) |
| CLP-1 | mannose 2.78%, galacturonic acid 4.07%, glucose 88.62%, galactose 1.80% and arabinose 2.39% | (65) |
| CLP2 | rhamnose 5.79%, galacturonic acid 7.78%, glucose 9.99% and galactose 13.30%. | (65) |
| CTP | rhamnose, mannose, glucose, and galactose | (66) |
| CDP-4 | straight-chain glucose | (66) |
| SPA | glucose and galactose are mainly composed of arabinose, rhamnose and mannose | (67) |
| - | α-l,4-D-glucan, α-L-arabino-3,6-β-D-galactan, pectic polysaccharides and 4-O-methyl-D-glucurono-D-xylan. | (68) |
| - | glucose, galactose, rhamnose, arabinose and fructose | (69) |

**Table 6.** Other compounds from Cistanche species

| **Compound category** | **Name** | **R** | **Ref** |
| --- | --- | --- | --- |
| Benzyl alcohol glycosides | salsaside A (Fig. 1a) | R1=H  R2=trans-Caf | (42) |
|  | salsaside B (Fig. 1a) | R1=trans-Caf  R2=H | (42) |
|  | salsaside C1 (Fig. 1a) | R1=p-trans-Cou  R2=H | (42) |
|  | salsaside C2 (Fig. 1a) | R1=p-cis-Cou  R2=H | (42) |
| Phenylacylated oligosugars | Cistanoside F (Fig. 1b) | R1=trans-Caf  R2=H | (70) |
|  | Cistanoside I (Fig. 1b) | R1=p-trans-Cou  R2=H | (27) |
|  | Cistantubulose A1/A2 (Fig. 1b) | R1=trans-Caf  R2=Glc | (31) |
|  | Cistansinensose A1/A2 (Fig. 1b) | R1=trans-Caf  R2=Rha | (40) |
| monoterpenoids | 8-hydroxygeraniol (Fig. 1c) | R=H | (47) |
|  | 8-hydroxygeraniol-1-β-D-glucopyranoside (Fig. 1c) | R=Glc | (71) |
|  | (2E,6R)-8-hydroxy-2,6-dimethyl-2-octenotic acid (Fig. 1d) | R=H | (72) |
|  | Kankanoside E (Fig. 1d) | R=Glc | (48) |
|  | (2E,6Z)-8-O-β-D-glucopyranoside-2,6-dimethyhyl-2,6-octadienoic acid (Fig. 1e) | R=Glc | (48) |
|  | (2E)-2,6-dimethyl-2,7-octadiene-1,6-diol (Fig. 1f) | R1=CH3  R2=CH2OH | （73) |
|  | (2Z)-2,6-dimethyl-2,7-octadiene-1,6-diol (Fig. 1f) | R1=CH2OH  R2=CH3 | (73) |
| Nitrogen-containing | Uridine | - | (45) |
|  | Inosine | - | (27) |
|  | 2’-O-methyladenosine | - | (27) |
|  | (3R)-3-hydroxy-1-methyl-2-pyrrolidinone | - | (45) |
|  | (3R)-3-hydroxy-2-pyrrolidinone |  | (45) |
|  | (2,5-dioxo-4-imidazolidinyl)-carbamic acid | - | (74) |
|  | Succinimide | - | (29) |
|  | 2-methanol-5-hydroxy-pyridine | - | (73) |
|  | betaine | - | (75) |

Caf: caffeoyl; Cou: coumaroyl; Glc: β-D-glucopyranosyl; Rha: α-L-rhamnopyranosyl

**Table 7.** Immunomodulatory mechanism of cistanche

| **Functional Ingredients** | **Cell**  **Line/Animal Model or Method** | **Occurring Mechanism or Effect** | **Evaluation of Research Findings** | **Ref** |
| --- | --- | --- | --- | --- |
| CPCD | modulator activities of CPCD on activating dendritic cells (DCs) and the adjuvant potential for foot and mouth disease vaccine (FMDV) | TLR-2 or TLR-4 antibodies suppressed levels of CPCD-mediated CD40 and CD86 as well as IL-6 and IL-1β in DCs. CPCD induced the phosphorylation of MAPKs related molecules and NF-κB. | CPCD could effectively stimulate stronger humoral and cellular responses by modulating DC activation through TLR-2/TLR-4 related MAPKs and NF-κB pathway. | (76) |
| AECCD(aqueous extracts of cultivated Cistanche deserticola) | ICR mice against ovalbumin (OVA), dendritic cells (DC) activation mechanism by AECCD | AECCD elicited vigorous and long-term IgG responses with mixed Th1/Th2 responses and up-regulated levels of Th-associated cytokines (CD4+IL-4, CD4+IFN-γ and CD8+IFN-γ). | AECCD could elicit potent and durable antigen specific im­ mune responses through DC activation. | (77) |
| PGC | Wistar female rats were selected. The left ovaries for all rats except in the blank control group(BC) were removed, and the right ovaries were removed in 80%. | PGC increase the activity, the organ index (thymus, spleen, uterus), E-2, T, BGP level in serum, beta-EP level in plasma, AR level in hypothalamus, ER level in hypothalamus, pituitary, uterus in perimenopausal model rats. And it also reduced FSH, LH, GnRH level in serum, and improved uterine and ovarian lesions in perimenopausal model rats. | Each dose of PCG could counteract the disorder of sex hormone in perimenopausal model rats, correct the imbalance of ER and AR level, enhance and restore the effect of uterus and the nerve cells of hypothalamic, and improve immune function. | (78) |
| WPCD | DCs from C57BL/6 mice, ovalbumin (OVA) (Sigma) was used as the model antigen and female ICR mice | WPCD significantly promoted the maturation and function of murine marrow-derived dendritic cells (BM-DCs) through up-regulating the expression levels of MHC-II, CD86, CD80, and CD40, allogenic T cell proliferation, and the yields of IL-12 and TNF-α via toll-like receptor4 (TLR4), as indicated by in vitro experiments. | WPCD could modulate immune responses in vitro and in vivo. | (79) |

**Table 8.** Neuroprotective effect mechanism of cistanche

| **Functional**  **Ingredients** | **Cell**  **Line/Animal Model or Method** | **Occurring Mechanism or Effect** | **Evaluation of Research Findings** | **Ref** |
| --- | --- | --- | --- | --- |
| Herba Cistanches | induced oxidative damage in MES23.5 cells using H2O2 | all drug-containing serums improved the survival rate of H2O2-injured MES23.5 cells, inhibited pro-apoptotic FasL and caspase-3 expression, promoted anti-apoptotic Bcl-2 expression. | Chinese medicines used to tonify the kidney can protect nerve cells by regulating the expression of apoptosis-related factors and neurotrophic factors in MES23.5 cells. | (80) |
| ECA | kainic acid-induced seizures in rats | Rats pre injected with Echinacea could reduce the effect of kainic acid on glutamate concentration in mice, slow down neuron loss and microglia activation, and inhibit the expression of proinflammatory cytokine genes in hippocampus | echinacoside is the potentially useful in the prevention of epilepsy | (81) |
| CDP | PC12 cell model | CDP (0.05, 0.5 and 5 mu g/ml) attenuated PC12 cell death, preserved MMP and calcium homeostasis; inhibited oxidative stress and decreased cell apoptosis. Moreover, CDP (5 mu g/ml) markedly stimulated DJ-1 secretion and expression. | CDP exerts neuroprotective effect against OGD/RP-induced injury by inhibiting oxidative stress and regulating the DJ-1 pathway | (82) |
| PhG | Parkinson's mouse model induced by neurotoxin 1-methyl-4-phenyl-1,2,3,6-tetrahydropyridine (MPTP) | neuroprotective effects of PhGs on nigral dopaminergic neurons were confirmed by the results of immunohistochemical staining. | PhG has neuroprotection | (83) |

**Table 9.** Antioxidant effect mechanism of cistanche

| **Functional**  **Ingredients** | **Cell**  **Line/Animal Model or Method** | **Occurring Mechanism or Effect** | **Evaluation of Research Findings** | **Ref** |
| --- | --- | --- | --- | --- |
| Cistanche deserticola | senescence accelerated OXYS rats | the oxidation rate of tryptophan and kynurenine in mice supplemented with Cistanche deserticola slowed down | Cistanche deserticola can slow down the development of cataract | (84) |
| cistanche | sevoflurane-induced aged cognitive dysfunction rat model | Cistanche deserticola can reduce oxidative stress by reducing nitrite and MDA and increasing SOD and CAT activities at the same time. | Cistanche deserticola can activate PPAR- γ Signal transduction plays an antioxidant role in the development of sevoflurane induced cognitive dysfunction | (85) |
| PhG | Kidney yang deficiency model | the contents of SOD and MDA in treatment group were higher than those in other groups，the Level of Hormone (T and E2). were increasing | PhG can restore the level of neutral hormones in the kidney yang deficiency model and improve the antioxidant effect | (86) |
| PhG | AD senescence accelerated mouse prone 8 (SAMP8) model | PHG significantly increased the density of dendritic spines in hippocampal CA1 region, accompanied by increased expression levels of synaptophysin (SYN) and postsynaptic density 95 (PSD-95), decreased MDA content, and increased SOD and GSH PX activities | the ability of PhG to ameliorate cognitive deficits in SAMP8 mice may be related to promotion in synaptic plasticity involving antioxidant processes | (87) |

**Table 10.** Antitumor effect mechanism of cistanche

| **Functional**  **Ingredients** | **Cell**  **Line/Animal Model or Method** | **Occurring Mechanism or Effect** | **Evaluation of Research Findings** | **Ref** |
| --- | --- | --- | --- | --- |
| CTPG | HepG2 and BEL-7404 hepatocellular carcinoma (HCC) cells, H22 tumor mouse model | CTPG significantly inhibited the growth of HepG2 and BEL-7404 cells through the induction of cell cycle arrest and apoptosis, which was associated with the activation of MAPK  pathways characterized by the up-regulated phosphorylation of p38, JNK, and ERK1/2 and mitochondria-dependent pathway characterized by the reduction of mitochondrial membrane potential. The release of cytochrome c and the cleavage of caspase-3, -7, -9, and PARP were subsequently increased by CTPG treatment. CTPG combined with cisplatin further inhibited the growth of H22 cells and reduced the side effects  of cisplatin. | CTPG inhibited the growth of HCC through direct antitumor effect and indirect immunoenhancement effect, and improved the antitumor efficacy of cisplatin | (88) |
| CTPG | H22 cells； tumor mouse model established using male Kunming mice | CTPG treatment significantly suppressed H22 cell growth in a dose and time dependent manner；significantly increased Bax/ Bcl-2 ratio, reduced Δψm and enhanced the release of cytochrome c； the levels of cleaved caspase-8 and caspase-9 in both extrinsic and intrinsic signaling pathways were significantly increased that sequentially activated caspase-7 and -3 to cleave PARP. | CTPG suppressed H22 cell growth through both extrinsic and intrinsic apoptosis pathways | (89) |
| CTPG-W | Eca‑109 cells | CTPG‑W significantly reduced the viability of Eca‑109 cells through the induction of apoptosis and cell cycle arres； and the levels of cytochrome c and c‑Jun NH2 ‑terminal kinase were increased, which upregulated the levels of cleaved‑poly (ADP‑ribose) polymerase and cleaved‑caspase‑3, ‑7 and ‑9, but not caspase‑8. | CTPG‑W induced apoptosis of Eca‑109 cells through a mitochondrial‑dependent pathway | (90) |
| ECH | SW1990 pancreatic adeno-  carcinoma cells | ECH can markedly inhibit the proliferation of pancreatic adenocarcinoma cells by inducing the production of reactive oxygen species and the perturbation of mitochondrial membrane potential and thus triggering apoptosis; and ECH represses tumor cell growth through modulating MAPK activity | ECH inhibits cancer development | (91) |

**Table 11.** Hepatoprotective effect mechanism of cistanche

| **Functional**  **Ingredients** | **Cell**  **Line/Animal Model or Method** | **Occurring Mechanism or Effect** | **Evaluation of Research Findings** | **Ref** |
| --- | --- | --- | --- | --- |
| PhG | D-galactosamine (D-GalN)/lipopolysaccharide (LPS)-induced liver injury in mice | 20-acetylacteoside and tubuloside A inhibited D-GalN-induced death of hepatocytes，isolates and cistantubuloside B1 also reduced TNF-a-induced cytotoxicity in L929 cells | fresh cistanche extract has a protective effect on the liver | (92) |
| PhGs | NADPH/CCI4-induced lipid peroxidation in rat liver microsomes | the tested four phenylethanoids consecutively inhibited both hepatocytes lipid peroxidation and AST release to the medium and alleviated the cell death induced by CCI4 | phenylethanoids were potent hepatoprotective agents against CCI4 intoxication. | (93) |
| Acteoside (ACT) | GalN/LPS-induced-acute-hepatic-injury mouse model | GalN/LPS administration markedly increased MPO levels in liver tissues, indicating significant macrophage infiltration, whereas the CA, HT, and 3-HPP pretreatments reduced the levels of MPO; the ACT group reduced levels of HMGB1, TNF-α, and IL-6 | ACT metabolites could be responsible for the potent hepatoprotective activity as well as the other therapeutic eﬀects. | (94) |
| CDP-C | Hep G2 and Er Guo-tou white spirit was used to establish liver injury model in ICR mice | In vitro research, CDP-C promoted viability of HepG2 cells；CDP-C can reduce the contents of MDA and TG in liver, and modulate the enzyme activities | CDP-C can be used in the treatment of alcoholic liver disease | (95) |

# Supplementary Figures and Tables

**（a）** **（b）**

**(c)** **(d)**

**(e)** **(f)**

**Figure 1** Some chemical structures in cistanche


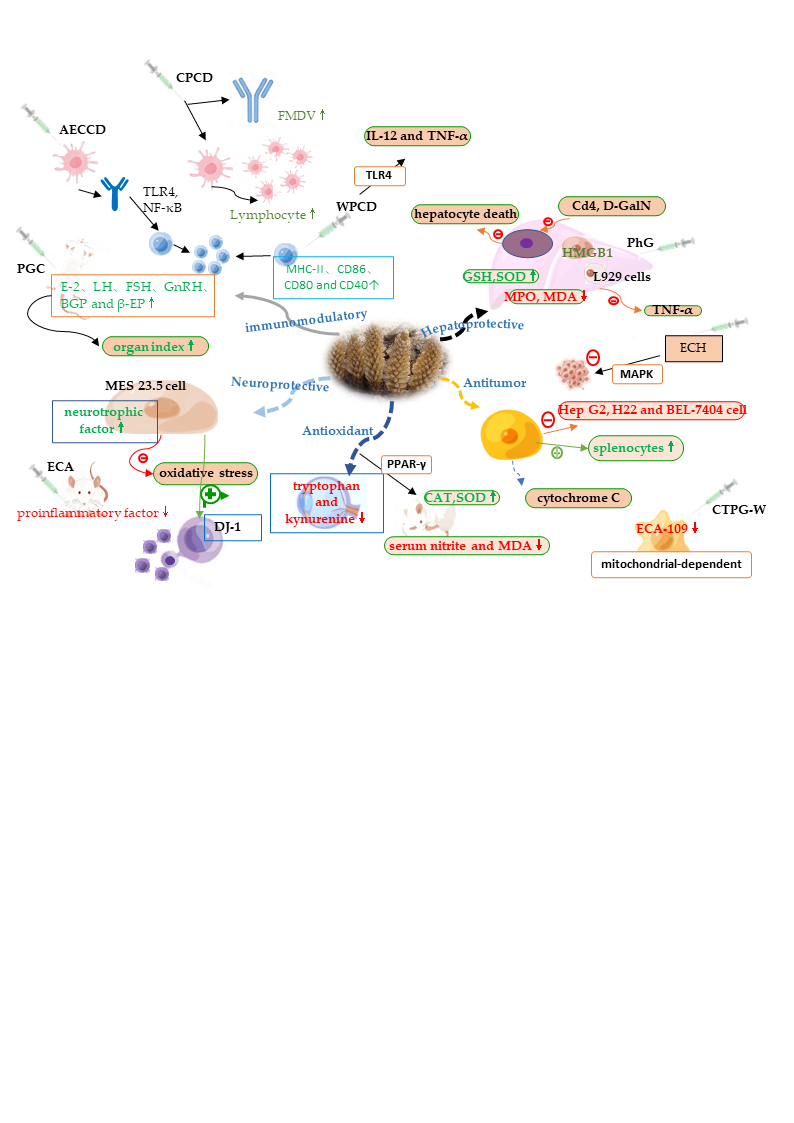


**Figure 2.** Immunomodulatory effect mechanisms of cistanche
